# Continuous observations of the surface energy budget and meteorology over the Arctic sea ice during MOSAiC

**DOI:** 10.1038/s41597-023-02415-5

**Published:** 2023-08-04

**Authors:** Christopher J. Cox, Michael R. Gallagher, Matthew D. Shupe, P. Ola G. Persson, Amy Solomon, Christopher W. Fairall, Thomas Ayers, Byron Blomquist, Ian M. Brooks, Dave Costa, Andrey Grachev, Daniel Gottas, Jennifer K. Hutchings, Mark Kutchenreiter, Jesse Leach, Sara M. Morris, Victor Morris, Jackson Osborn, Sergio Pezoa, Andreas Preußer, Laura D. Riihimaki, Taneil Uttal

**Affiliations:** 1 National Oceanic and Atmospheric Administration (NOAA) Physical Sciences Laboratory (PSL), Boulder, Colorado USA; 2 Cooperative Institute for Research in Environmental Sciences (CIRES), University of Colorado, Boulder, Colorado USA; 3 School of Earth and Environment, University of Leeds, Leeds, UK; 4 Atmospheric Dynamics and Analytics Branch, DEVCOM Army Research Laboratory, White Sands, New Mexico USA; 5 College of Earth, Ocean, and Atmospheric Sciences, Oregon State University, Corvallis, Oregon USA; 6 National Oceanic and Atmospheric Administration (NOAA) Global Monitoring Laboratory (GML), Boulder, Colorado USA; 7 Pacific Northwest National Laboratory (PNNL), Richland, Washington USA; 8 Alfred Wegener Institute, Helmholtz Centre for Polar and Marine Research, Bremerhaven, Germany

**Keywords:** Cryospheric science, Atmospheric science

## Abstract

The Multidisciplinary drifting Observatory for the Study of Arctic Climate (MOSAiC) was a yearlong expedition supported by the icebreaker *R/V Polarstern*, following the Transpolar Drift from October 2019 to October 2020. The campaign documented an annual cycle of physical, biological, and chemical processes impacting the atmosphere-ice-ocean system. Of central importance were measurements of the thermodynamic and dynamic evolution of the sea ice. A multi-agency international team led by the University of Colorado/CIRES and NOAA-PSL observed meteorology and surface-atmosphere energy exchanges, including radiation; turbulent momentum flux; turbulent latent and sensible heat flux; and snow conductive flux. There were four stations on the ice, a 10 m micrometeorological tower paired with a 23/30 m mast and radiation station and three autonomous Atmospheric Surface Flux Stations. Collectively, the four stations acquired ~928 days of data. This manuscript documents the acquisition and post-processing of those measurements and provides a guide for researchers to access and use the data products.

## Background & Summary

The Arctic is increasingly characterised by seasonal coverage of sea ice and, in areas where sea ice persists through the summer, the age of the ice has decreased^1^. These changes are linked with other environmental shifts in the coupled system with implications for regional and global climate. Upon this backdrop, the societal importance of the Arctic is growing. Information services, including climate projections, weather forecasting, and seasonal outlooks are needed to support safety and sustainability in the region. To this end, the Multidisciplinary drifting Observatory for the Study of Arctic Climate (MOSAiC) conducted a yearlong drifting scientific expedition based on and around the icebreaker *R/V Polarstern*^2^ between October 2019 and October 2020 to document the annual cycle of coupled atmosphere, sea ice, and ocean processes in the central Arctic^3–5^. Collecting data to understand the thermodynamic and dynamic physics that modulate ice growth, melt, and motion was a priority. A collaborative team led by the University of Colorado and the NOAA Physical Sciences Laboratory (PSL), and including the University of Leeds and the U.S. Department of Energy (DOE) Atmospheric Radiation Measurement (ARM) Program^6^, observed near-surface meteorology and variables comprising the surface energy budget (SEB).

MOSAiC’s design lineage begins with Fridtjof Nansen’s *Fram* expedition of 1893–1896 that demonstrated the existence of the Transpolar Drift^7^; however, it was not until later Norwegian studies that attempts were made to understand the SEB over sea ice (e.g., ref. ^8^). Modern scientific cruises on icebreakers with ship-based measurements are somewhat routine, in particular during summer. Occasionally, observations related to the SEB are made (e.g., ref. ^9,10^). More comprehensive multidisciplinary studies that venture deeper into the pack ice are less common, and those featuring ice stations where *in situ* SEB observations are collected unhindered from vessels are rare. The Russian Federation carried out 41 North Pole drifting stations between 1933 and 2015^11^, some of which included observations of SEB terms. The Surface Heat Budget of the Arctic Ocean (SHEBA) experiment^12^ collected a full year of SEB measurements from multiyear ice in the Beaufort and Chukchi Seas in 1997–1998^13^, whereas the Arctic Ocean Experiment (AOE-2001)^14^, Arctic Summer Cloud Ocean Study (ASCOS)^15^, Arctic Clouds in Summer Expedition (ACSE/SWERUS 2014)^16^, and SWEDARCTIC/AO2018^17^ collected such measurements over the central Arctic Ocean in the summers of 2001, 2008, 2014, and 2018. Winter and spring observations were made in the western Eurasian basin by the Norwegian Young Sea Ice (N-ICE2015) experiment^18,19^ and in the Beaufort Sea during the Canadian Arctic Shelf Exchange Study (CASES)^20^. MOSAiC is the first study to make comprehensive SEB measurements while following the life cycle of an ice floe through the Transpolar Drift. The observations described here document the annual cycle of near-surface meteorology and SEB terms associated with ice floes comprising a mixture of first- and second-year ice, ice conditions characteristic of the New Arctic. MOSAiC was not only comprehensive and interdisciplinary, it was also unique in its spatial coverage, featuring assets distributed in an array across 10 s of km.

Observations of air-surface energy exchanges, meteorology, and surface properties were made on the sea ice in the MOSAiC Central Observatory (CO) near *Polarstern* at an installation called “Met City,” which included a 10-m micrometeorological tower, a 23–30 m telescoping mast, and a radiation station. Additionally, comparable observations were collected using three mobile Atmospheric Surface Flux Stations (ASFS), which were operated at the MOSAiC Distributed Network (DN) “L-sites” and for targeted experiments of opportunity within the CO. Measurements included meteorology, snow depth, sub-surface conductive heat flux, upward and downward broadband longwave and shortwave radiative fluxes, narrow-band infrared surface brightness temperature, 3-dimensional winds using ultrasonic anemometers, position and heading from GPS, and CO_2_/H_2_O gas densities using gas analysers. From these, fluxes of sensible heat, latent heat, and momentum were derived. Collectively, amongst the four stations, ~928 days of data were acquired and have been archived for community use. Here, we describe how these observing assets were deployed as well as how revisions were made in response to evolving logistical and environmental conditions. We also describe the data processing, quality control methodology, corrections, derivation of the fluxes, and uncertainties. Multiple data sets are archived, including raw and increasingly-refined levels of data to facilitate a range of users and applications.

## Methods

### Objectives

**Fig. 1:**
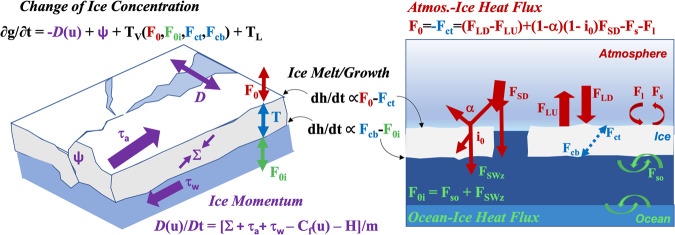
Schematic and equations of energy transfers affecting sea ice. Dynamic terms are in purple; thermodynamic terms at the surface-atmosphere interface in red (the subject of the present data set), through the ice in blue, and in the ocean in green. D = ice divergence; ψ is mechanical ice redistribution function (ridging); T is thermodynamic ice growth/melt rate; L is lateral melt; u is horizontal ice velocity; τ_a_ is wind stress; τ_w_ is ocean stress; Σ is force due to ice interaction; C_f_ is Coriolis force; H is gravitational acceleration down ocean surface slope; F_0_ is net atmosphere-ice heat flux, F_s_ is atmosphere sensible heat flux; F_l_ is latent heat flux; F_LD_ is incoming longwave (LW) flux; F_LU_ is outgoing LW flux; F_SD_ is incoming shortwave (SW) flux; α is the surface albedo; i_0_ = fraction of absorbed shortwave flux that penetrates into the ice; F_0i_ = net ocean-ice heat flux; F_so_ = ocean turbulent sensible heat flux; F_SWz_ is shortwave radiation reaching ice bottom; F_cb_, F_ct_ are the conductive heat fluxes at ice top and bottom, respectively; dh/dt = melt or growth of ice thickness.

One key objective of MOSAiC was to quantify the contributions of dynamic and thermodynamic processes to the mass balance of sea ice. Figure 1 schematizes the principal thermodynamic and dynamic energy terms that modulate the evolution of sea ice^21^. The dynamic terms (purple) include translational ice motion/divergence (D), drag from the ocean (τ_w_), wind stress on the ice/snow surface (τ_a_), and internal stress (Σ). The thermodynamics include the vertical distribution of energy in the atmosphere-ice-ocean column (T_V_), which is dominated by turbulence and radiation at the ice-atmosphere interface (reds), conduction through the snow and ice (blues), and mechanical transfer in the upper ocean (greens). Penetrating shortwave (i_0_) forms an extinction profile below the ice/snow surface affecting the distribution of absorption directly and the energy balance at the air-ice interface indirectly. Lateral thermodynamic transfer (T_L_) also occurs, most notably at breaks in surface type such as leads where differential horizontal heating occurs^22^ and amongst variable snow depth^23^.

At MOSAiC, an interdisciplinary collaboration was devised to achieve continuous observations of the terms in Fig. 1 with the dynamic component quantified by unified measurements at multiple locations. A staffed CO was based at *Polarstern* and surrounded by three intensive autonomous sites at distances of ~10–23 km, which were set amongst a larger array of GPS and less-instrumented stations up to distances of ~40 km, referred to as MOSAiC’s Distributed Network (DN)^24^. The thermodynamic observations within the ocean are reported by^25,26^, through the ice by^27,28^, and the ice dynamics experiment is described by^29^. The present work details the measurements at the air-ice/snow interface (reds in Fig. 1) suitable for solving the surface net energy equation (neglecting melt):

1 $${F}_{0}=-{F}_{ct}=\left({F}_{LD}-{F}_{LU}\right)+\left(1-\alpha \right)\left(1-{i}_{0}\right){F}_{SD}-{F}_{s}-{F}_{l},$$

where F_ct_ is the conductive flux at the snow/ice-atmosphere interface; F_LD_ and F_LU_ are the downward and upward directed longwave radiative fluxes; α is albedo; i_0_ is the fraction of the net shortwave flux that is absorbed below, rather than at the surface; F_SD_ is the downwelling shortwave flux; and F_s_ and F_l_ are the turbulent sensible and latent heat fluxes. Note that all terms in Eq. 1 are defined positive toward the interface except for F_s_ and F_l_, which are shown positive upwards, as is their typical convention; these are the conventions used for the data set. The objective of the project described here was to observe the terms comprising F_0_. F_SD_ and the reflected shortwave, F_SU_, were measured such that at the surface,

2 $$\left(1-\alpha \right)\left(1-{i}_{0}\right){F}_{SD}={F}_{SD}-{F}_{SU}.$$

In practice, F_ct_ was observed below rather than at the surface, and thus imbalances in Eq. 1 represent the storage of energy as it passes through the layer^30^ plus error.

Collaborative projects collected measurements suitable for deriving i_0_ as well as observations of F_ct_ to which our measurements are complementary^4,5^. Similarly, we observed several variables pertaining to ice dynamics, including geodetic information and wind stress on the surface, τ_a_. Further details on complementary measurements can be found in other publications, including a larger array of ice-profiling, above and below ice radiation^4^, ocean profiling^5^ and atmospheric profiling, cloud properties, aerosol, and trace gas measurements^3^, some of which were made at the DN as well^24^.

### Instrumentation and Operations

**Fig. 2:**
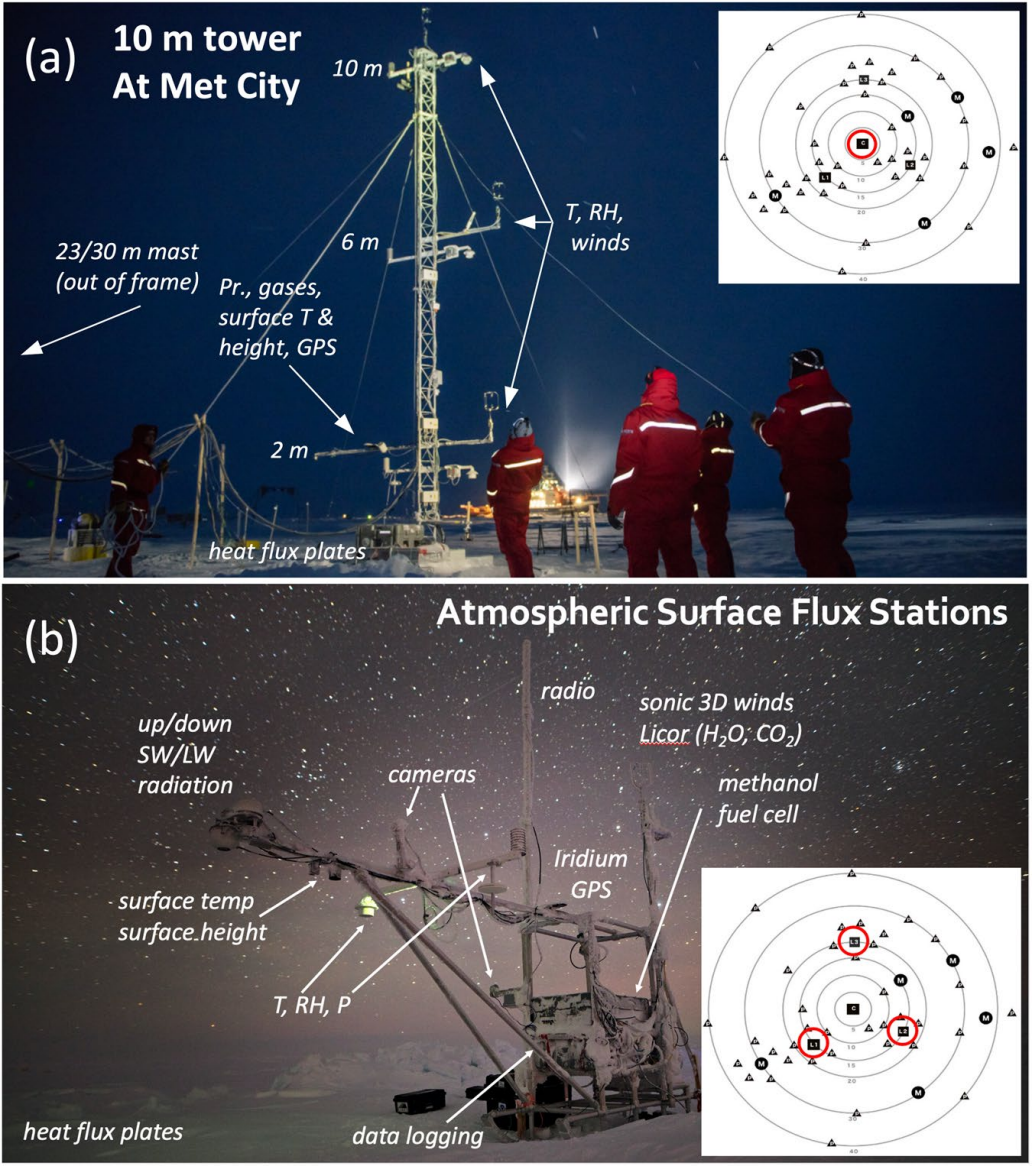
(**a**) Image of the tower at Met City in October 2019 (photo by Esther Horvath). (**b**) Image of ASFS-50 on 1 January 2020 (photo by Michael Gallagher). Both panels are annotated with instrument locations. The insets are maps from the DN at the beginning of Leg 1 with distances in km from *Polarstern* (large circles) (credit: Daniel Watkins). The red circles in (**a**) are the CO where the tower was positioned; the three red circles in (**b**) are the L-sites (L1 lower-left (ASFS-40), L2 (ASFS-30) lower-right, L3 (ASFS-50) top) where ASFS were deployed in October 2019.

To observe radiative and turbulent fluxes in Eq. (1), the instrument suites (Fig. 2) all included upward- and downward-facing broadband pyranometers (F_SD_, F_SU_) and pyrgeometers (F_LD_, F_LU_); ultrasonic anemometers measuring wind in three dimensions (for wind speed and direction, and for eddy covariance measurements of sensible heat flux, F_s_, and friction velocity, u_*_); open-path gas (H_2_O and CO_2_) analysers (for eddy covariance measurements of latent heat flux, F_l_); and conductive heat flux plates (F_ct_). These flux measurements were complemented by basic meteorology (atmospheric pressure, temperature, humidity, winds); localised snow depth measurements using sonic rangers; position and heading using GPS; and surface brightness temperature using infrared thermometers (IRT). Instrument selection was based on the experience developed from prior ship and drift campaigns in the sea ice^9,13^ as well as measurements made from land stations surrounding the Arctic^31–33^. The full list of sensors with other relevant details can be found in Table 1. We first describe the platforms and layout of the four stations followed by the theory of operation, measurement, and post-processing methods.

**Table 1:** List of sensors, variables, and uncertainties.

Instrument	Platform	Variable	Sampling Rate	Reported Avgs	Uncertainty	Valid Range	Notes
Vaisala PTU307	Tower, ASFS	Pressure	1 Hz (twr), 0.2 Hz (ASFS)	1 min, 10 min	0.15 hPa	one-sided (±)	total accuracy reported by manufacturer
Vaisala PTU307, HMT337	Tower, ASFS	Temperature	1 Hz (twr), 0.2 Hz (ASFS)	1 min, 10 min	0.4-0.3 C	one-sided (±)	accuracy reported by manufacturer. −40 to −10 C
Vaisala PTU307, HMT337	Tower, ASFS	Relative Humidity	1 Hz (twr), 0.2 Hz (ASFS)	1 min, 10 min	1.6–1.8%	one-sided (±)	reported by manufacturer @ 80–100% RH, −20 to + 40 C
Vaisala WXT530	Mast	Pressure	1 Hz	1 min, 10 min	1 hPa	one-sided (±)	accuracy reported by manufacturer
Vaisala WXT530	Mast	Temperature	1 Hz	1 min, 10 min	0.3 C	one-sided (±)	accuracy reported by manufacturer
Vaisala WXT530	Mast	Relative Humidity	1 Hz	1 min, 10 min	5%	one-sided (±)	accuracy reported by manufacturer @ 90–100% RH
Metek USA1	Mast	Wind Speed	10 Hz	1 min, 10 min	0.3 ms^−1^	max error	component error reported by manufacturer; structural interference not considered
Metek u-Sonic3 Cage MP	Tower, ASFS	Wind Speed	20 Hz	1 min, 10 min	0.3 ms^−1^	max error	component error reported by manufacturer; structural interference not considered
Metek USA1	Mast	Wind Direction	10 Hz	1 min, 10 min	~ 2–5°	max error	dominated by estimated mount alignment over measurement error.
Metek u-Sonic3 Cage MP	Tower, ASFS	Wind Direction	20 Hz	1 min, 10 min	~ 2°	max error	dominated by estimated mount alignment over measurement error.
Hukseflux SR30-D1	ASFS	F_SD_	0.2 Hz	1 min, 10 min	1.8%	95% CI	specificed for ISO 98-3 (see text for details): valid for clear-sky, SZA < 75°; spectral range: 285–2800 nm
Hukseflux SR30-D1	ASFS	F_SD_ (diffuse)	0.2 Hz	1 min, 10 min	3.1 Wm^−2^	95% CI	specificed for ISO 98-3 (see text for details); spectral range: 285–2800 nm
Hukseflux SR30-D1	ASFS	F_SU_	0.2 Hz	1 min, 10 min	3.1 Wm^−2^	95% CI	assumed equal to F_SD_ diffuse; spectral range: 285–2800 nm
Hukseflux IR20	ASFS	F_LD_	0.2 Hz	1 min, 10 min	2.6 Wm^−2^	±1σ	empirical, Cox *et al*.^33^, ref therin: mean value ~4 Wm^−2^ (clear), 1 Wm^−2^ (cloudy); spectral range: 4.5–40 *μ*m
Hukseflux IR20	ASFS	F_LU_	0.2 Hz	1 min, 10 min	1 Wm^−2^	±1σ	following cloudy sky F_LD_ value from Cox *et al*.; approx. using cloudy value for F_LD_; spectral range: 4.5–40 *μ*m
Eppley PSP	ICERAD	F_SD_	1 Hz	1 min, 10 min	2.4%	95% CI	specificed for ISO 98-3 (see text for details): valid for clear-sky, SZA < 75°; spectral range: 285–2800 nm
Eppley PSP	ICERAD	F_SD_ (diffuse)	1 Hz	1 min, 10 min	4.5 Wm^−2^	95% CI	specificed for ISO 98-3 (see text for details); spectral range: 285–2800 nm
Eppley PSP	GNDRAD	F_SU_	1 Hz	1 min, 10 min	4.5 Wm^−2^	95% CI	assumed equal to F_SD_ diffuse; spectral range: 285–2800 nm
Eppley PIR	ICERAD	F_LD_	1 Hz	1 min, 10 min	2.6 Wm^−2^	±1σ	empirical, Cox *et al*.^33^, ref therin; spectral range: 4–50 *μ*m
Eppley PIR	GNDRAD	F_LU_	1 Hz	1 min, 10 min	1 Wm^−2^	±1σ	following cloudy F_LD_ value from Cox *et al*.^33^; spectral range: 4–50 *μ*m
Metek uSonic-3 & USA-1	Tower, ASFS	F_s_ (eddy cov)	20 Hz	10 min	4.8 Wm^−2^	RMSE	sensor intercomparison
Licor 7500-DS	Tower, ASFS	F_l_ (eddy cov)	20 Hz	10 min	50%	N/A	Persson *et al*.^13^
various inputs	Tower, ASFS	F_s_ (bulk)	10 min	10 min	5.8 Wm^−2^	RMSE	randomized errors to inputs; −20 C, −1 C gradient, 10 ms^−1^
various inputs	Tower, ASFS	F_l_ (bulk)	10 min	10 min	43%	RMSE	randomized errors to inputs; −20 C, +1 C gradient, 10 ms^−1^
Hukseflux HFP01	Tower, ASFS	F_CT_	1 Hz (twr), 0.2 Hz (ASFS)	1 min, 10 min	7–14%	95% CI	Sauer *et al*.^60^; specified for ISO 98-3, range of k from Table 2, Sturm *et al*.^91^
Hemisphere V102 GPS	Tower, ASFS	heading	1 Hz (twr), 0.2 Hz (ASFS)	1 min, 10 min	0.75°	RMSE	accuracy reported by manufacturer
Hemisphere V102 GPS	Tower, ASFS	position	1 Hz (twr), 0.2 Hz (ASFS)	1 min, 10 min	1.2 m	RMSE	accuracy reported by manufacturer
Apogee IRT	Tower, ASFS	Brightness Temp.	1 Hz (twr), 0.2 Hz (ASFS)	1 min, 10 min	0.12–0.14 C	95% CI	uncertainty range reported by calibration certificates; spectral range 8–14 *μ*m
SR50	Tower, ASFS	surface height	1 Hz (twr), 0.2 Hz (ASFS)	1 min, 10 min	1.2 cm	±2σ	manufacturer report & est. error from air temp

In the CO at “Met City” a 10 m micrometeorological tower was deployed. The tower frame had a small footprint (0.34 m triangle), was climbable, freestanding, and could be raised/lowered by hand with a small team by tipping from a hinged base. It was instrumented at three levels - nominally 2, 6, and 10 m - with meteorological instruments and sonic anemometers. The gas analyser, GPS, infrared thermometer, and acoustic ranger were mounted on the 2 m boom. The gas analyser was initially mounted on the 6 m boom for 8 days in October 2019 before being lowered to 2 m for easier cleaning access during winter. It was returned to 6 m during summer 2020. Two conductive flux plates were installed near the tower, with one located south of the tower and the second to the north underneath the infrared thermometer and acoustic ranger. Radiative fluxes were measured nearby by a collaborative project supported by the DOE-ARM Program (ARM Mobile Facility 2, AMF2)^34^. The upward-looking measurements were made from approximately 1 m height, while the downward-looking measurements were made from approximately 3 m height. A telescoping mast topped with meteorological measurements and a sonic anemometer was raised nearby to 30 m (and later lowered to 23 m) to extend observations deeper into the atmospheric boundary layer (ABL). Collaborative projects also instrumented the 10-m tower with particle counters for aerosols and blowing snow along with inlets for trace gas sampling conducted in a nearby hut (see ref. ^3^).

A heated box was positioned at the base of the tower containing a Campbell Scientific CR1000X data logger and a ruggedized PC to perform the data acquisition. The box was network connected (wired or wireless at different times) to data ingest and management computers aboard *Polarstern*. During autumn and winter, this box also operated the mast instrumentation, but later in spring the mast was operated by a second, redundant system when the tower and mast drifted apart on separate ice floes. The PC at the 10 m tower allowed low-latency, high-precision time stamping and enabled measurements to continue uninhibited if communications were lost as long as power could be supplied. A power hub was positioned at Met City to deliver electricity from *Polarstern*, and several gas generators were stored on site to supply backup power when needed. Other atmosphere-focused projects at Met City sampled precipitation and remotely-sensed the ABL structure (see ref. ^3^). Ice and ocean measurements by collaborative teams described in the previous section were also made at Met City.

To carry out mobile autonomous observations, comparable to those at Met City but in a scaled-down integrated platform, three ASFS were developed. The ASFS were metal pipe structures featuring a 3 m horizontal boom positioned at 2 m height and a 3 m vertical mast for mounting instruments, all set on custom polytetrafluoroethylene (PTFE) skis and configured similarly to a rigid Nansen sled. A large weathertight box contained electronics, power distribution and regulation, communications, and data logging (CR1000X) equipment. Each station was powered using an EFOY Pro 2400 Duo direct methanol fuel cell fuelled by two 60 L containers of methanol located in a separate storage box on the sled. The device is rated for 110 W while the ASFS operated nominally on 60–70 W, although basic operations could be sustained at ~30 W. The station could also be modified for AC power. The system could operate autonomously with more than 2 months of fuel, but each station was visited every 1–6 weeks when not operated at staffed locations. With instrumentation removed, each sled weighed approximately 350 kg, and was pushed manually, towed by snowmobile, or transported by sling load suspended from a helicopter or crane. The primary means of communication was by 2.4 GHz radio modem, which required line-of-sight < ~15 km distance to a base station at *Polarstern*. Secondary communications were through a peer-to-peer Iridium connection. Relatively large data volumes were collected (~120 mb/day/station), which could be received in real time via radio. Only a small subset could be transferred by satellite. Because remote data transfers could not be guaranteed, all data were stored locally on 16 GB microSD cards, which were exchanged during each maintenance visit. Identification numbers 30, 40, and 50 were assigned to the ASFS and are used to distinguish the stations in the data files and henceforth in this manuscript.

### Evolving spatial distribution of assets

**Fig. 3:**
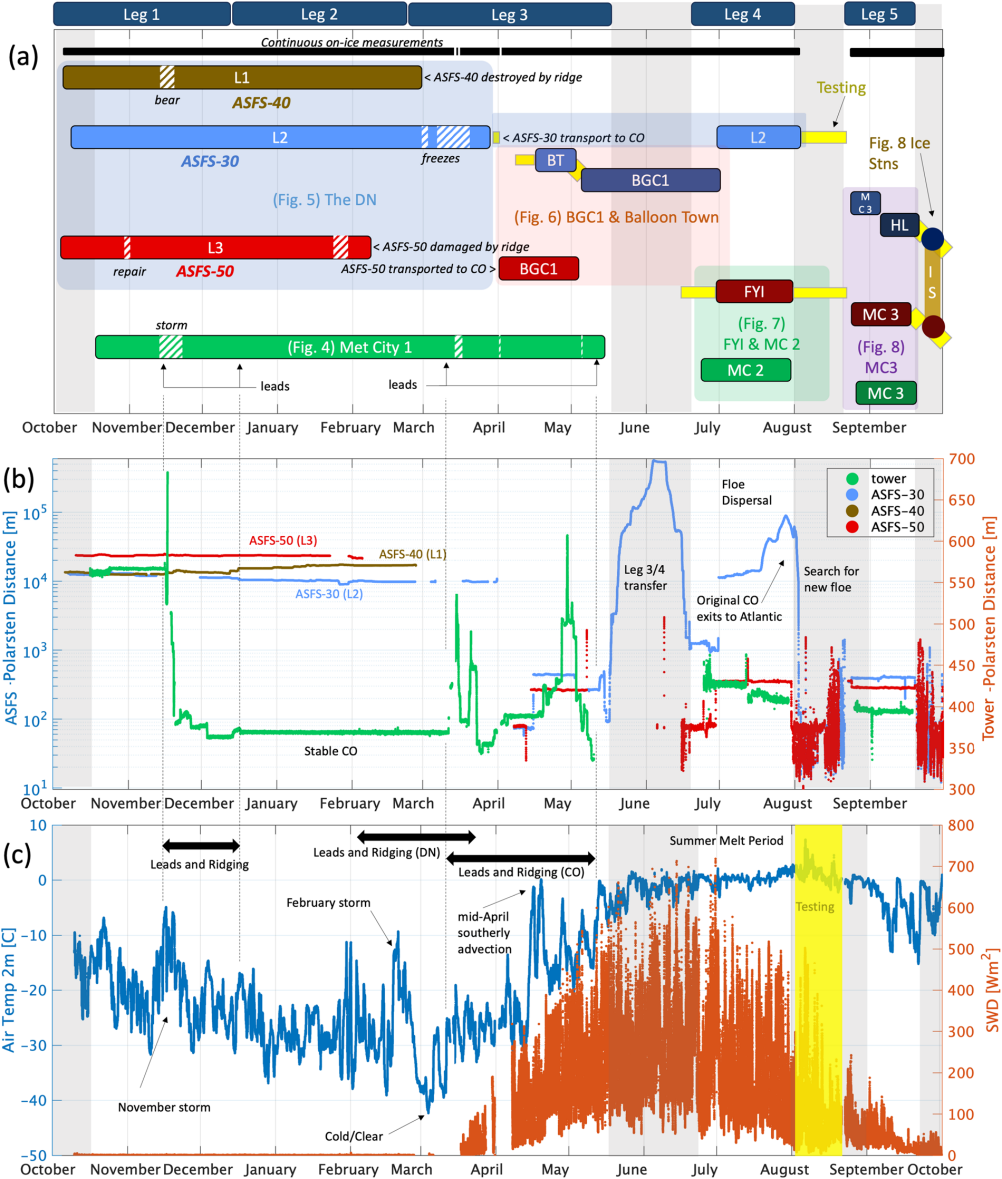
(**a**) Timeline of installations. “Legs” shown along the top refer to the five legs of the expedition; for precise dates, refer to ref. ^3^. Hatch patterns are missing or intermittent data. The vertical grey bars are periods when *Polarstern* was underway. The horizontal yellow bars are periods of testing and have data of limited scientific use. The black horizontal bar denotes the periods when data were continuous in the aggregate. In (**a**), the Figures in parentheses illustrate the setup during these time periods, which are described individually under section *Observation context and user of assets*. MC is a shorted form of “Met City” and the numbers 2 and 3 denote the second and third deployments of Met City, which were at different locations than the original (see main text). (**b**) Distances between *Polarstern* and each ASFS (left axis, log scale) and the tower (right axis, linear scale). (**c**) Composite (tower/all ASFS) temperature (left axis) and shortwave downwelling radiation (F_SD_, right axis). The vertical yellow bar marks when *Polarstern* was underway in search of a new floe and no scientific data were collected from the ice. The data shown during this period is from testing of ASFS-50 carried out on the aft deck of *Polarstern*: they are not considered scientifically valid and are only shown here for reference.

MOSAiC included a staffed CO and an unstaffed DN (insets, Fig. 2). Working in two teams based on *Polarstern* at the CO and *Akademik Fedorov* within the DN^24^ during October 2019, the tower was deployed at Met City in the CO and the ASFS at three L-sites in the DN surrounding the CO in roughly a triangular pattern at distances of ~13 km (ASFS-30 & 40) and ~23 km (ASFS-50). All four ice floes serving as station platforms had formed in late 2018 on the East Siberian Shelf and were entering their second winter^35^. The DN installations were set up within 60–200 m of the floe edges on ice of varying thickness, with L1 (ASFS-40) placed on the thickest ice and L3 (ASFS-50) on the thinnest ice (see ref. ^24^ for further details). Data were collected from early October 2019 through September 2020 during five time periods (“Legs”) separated by resupply and staffing exchanges, including two redeployments of Met City between Legs 3/4 and Legs 4/5 (Fig. 3a,b). The CO/DN spatial configuration remained relatively stable for most of the array’s drift before it exited to the Greenland Sea in July 2020, but the initial planned configuration of Met City and the three ASFSs ended towards the latter part of Leg 2 when ice dynamics events damaged two of the ASFS (Fig. 3b). The scientific use of the surviving two ASFS during later Legs had diverse objectives (described below), with deployments often within the CO.

Collectively, a rich, diverse documentation of the annual cycle was made. Here, to orient users to the complexity of the time series, we have divided the experiment into multiple periods depicted in the timeline in Fig. 3a,each of which is given a narrative description in the subsections that follow. To provide context, Fig. 3b shows distances between each station and *Polarstern*, and station-composites of air temperature and F_SD_ appear in Fig. 3c.

#### Met City 1

**Fig. 4:**
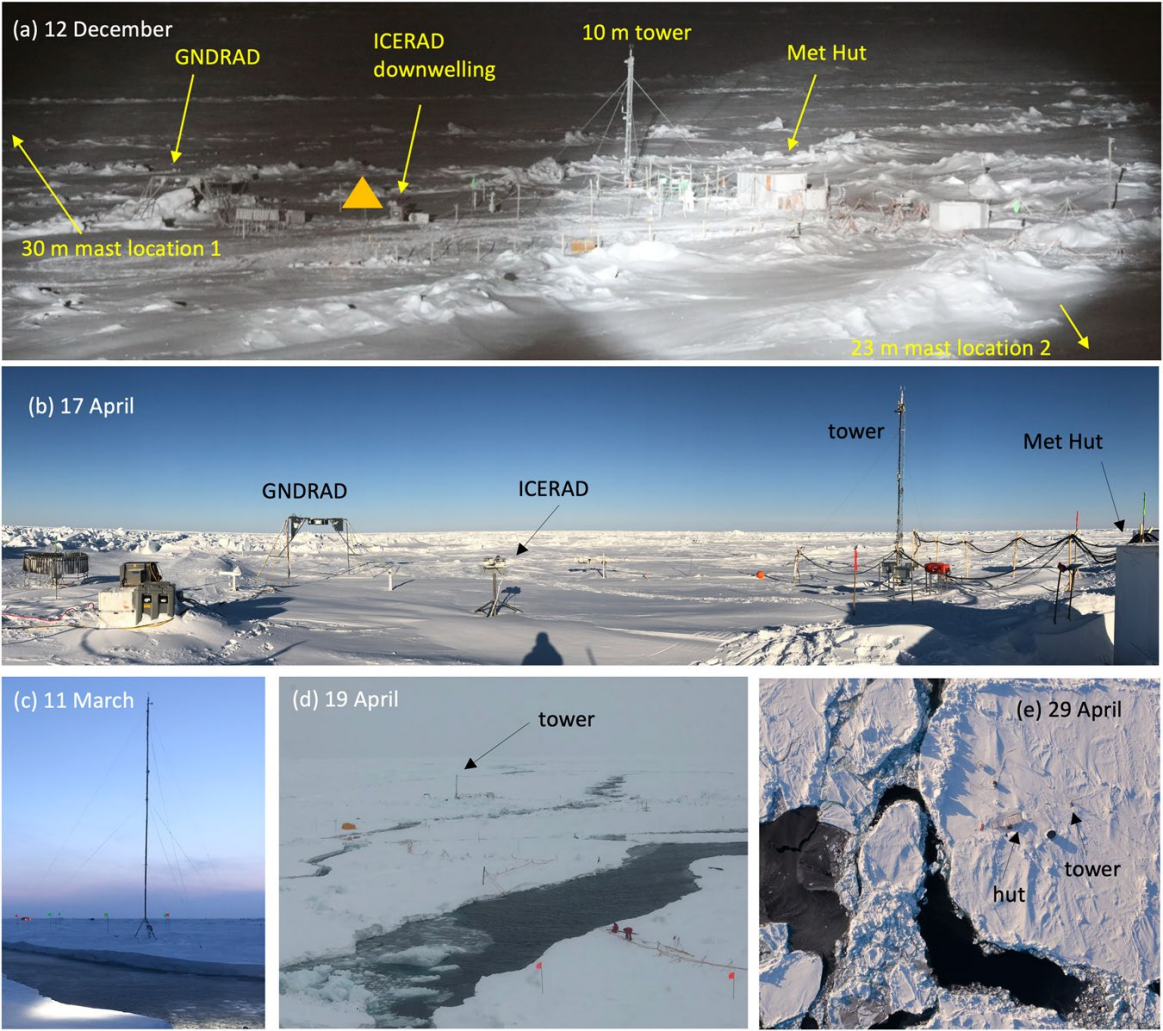
Photos of installations associated with Met City 1 (15 October 2019 – 10 May 2020). (**a**) Original configuration during Leg I after the November storm lead and ridging during which the 30-m mast fell, was rebuilt as 23-m mast, and moved from location 1 to location 2. The orange triangle denotes the approximate position of GNDRAD after it was moved on 17 March. Locations of other installations at Met City are listed for reference. (**b**) Configuration after GNDRAD and ICERAD were moved on 17 March. (**c**) 23-m mast at location 2. (**d,e**) Lead activity between *Polarstern* and Met City. Photo credit for (**e**) is ©UFA Show&Factual (unpublished, used with permission).

Met City (Fig. 4) was initially installed ~500 m from *Polarstern* along the outer edge of a pressure ridge colloquially known as the Fortress. Other installations, as well as power and fibre-optic connections to *Polarstern*, were positioned between Met City and the vessel so as to minimise the wind sector impacted by station activities. The impacts of local obstructions on turbulent flux measurements have been identified primarily in direction sectors towards the triangular tower frame itself, the Met Hut, and *Polarstern*. The associated sector data editing is described in a later subsection. The tower (Fig. 4a,b) and mast (Fig. 4a,c) were installed on approximately 1 m thick sea ice entering its second year, and specifically in areas that had not been previously ponded. The surrounding area also included a significant amount of ~30 cm ice in areas that had previously been melted-through melt ponds or open ocean. From 15–24 October, the tower was operated horizontally for intercomparison among its instrumented levels, after which the tower was raised. The mast sensors began data collection on 19 October mounted on or next to the prone tower, and they were then mounted on top of the 30 m mast as it was raised ~100 m in the direction of *Polarstern* from the tower at location 1 (denoted in Fig. 4a) on 26 October. Ice movement on 17 November cut the power supply to Met City and the station was operated discontinuously using generators until 28 November. This event also resulted in the collapse of the mast on 18 November, which was reinstalled at location 2 to a height of 23 m on 8–9 December after its sonic anemometer was repaired and tested. These ice dynamics events, and many additional events throughout the campaign, resulted in horizontal translations of the ice floes that significantly changed the relative bearing and distance between Met City and other installations, in particular *Polarstern* (Fig. 3b). For reference, the relative bearing and distance between *Polarstern* and Met City are calculated and archived in the data set.

For Met City 1, areas in the direction away from the CO (west-northwest through south to east-southeast in Leg 1 prior to the CO’s translation across the pole) featured relatively thin (30–50 cm) and smooth ice. This configuration remained largely unchanged until 11 March, when a lead (Fig. 4c) formed between the tower and the mast necessitating a controlled take-down of the 23 m mast. Met City remained separated from the CO/*Polarstern* thereafter and regularly experienced alternate periods of ridging and lead formation within a working shear zone through mid-May, incrementally reducing the size of the Met City floe (Fig. 4d,e). The tower was run on gas generators throughout most of this period. The reduced power resources resulted in occasional gaps of several hours in data for power-related repairs. Installations, including the ARM radiation/albedo rack (ICERAD/GNDRAD), were repositioned on 17 March closer to the tower, away from encroaching ridges and leads (see yellow triangle, Fig. 4a and new position Fig. 4b). Met City was decommissioned in the hours before a storm on 12 May largely destroyed the remainder of its ice floe. From mid-March to mid-April, the wind direction was primarily from the north, the same sector as *Polarstern*’s position relative to Met City.

#### Distributed network

**Fig. 5:**
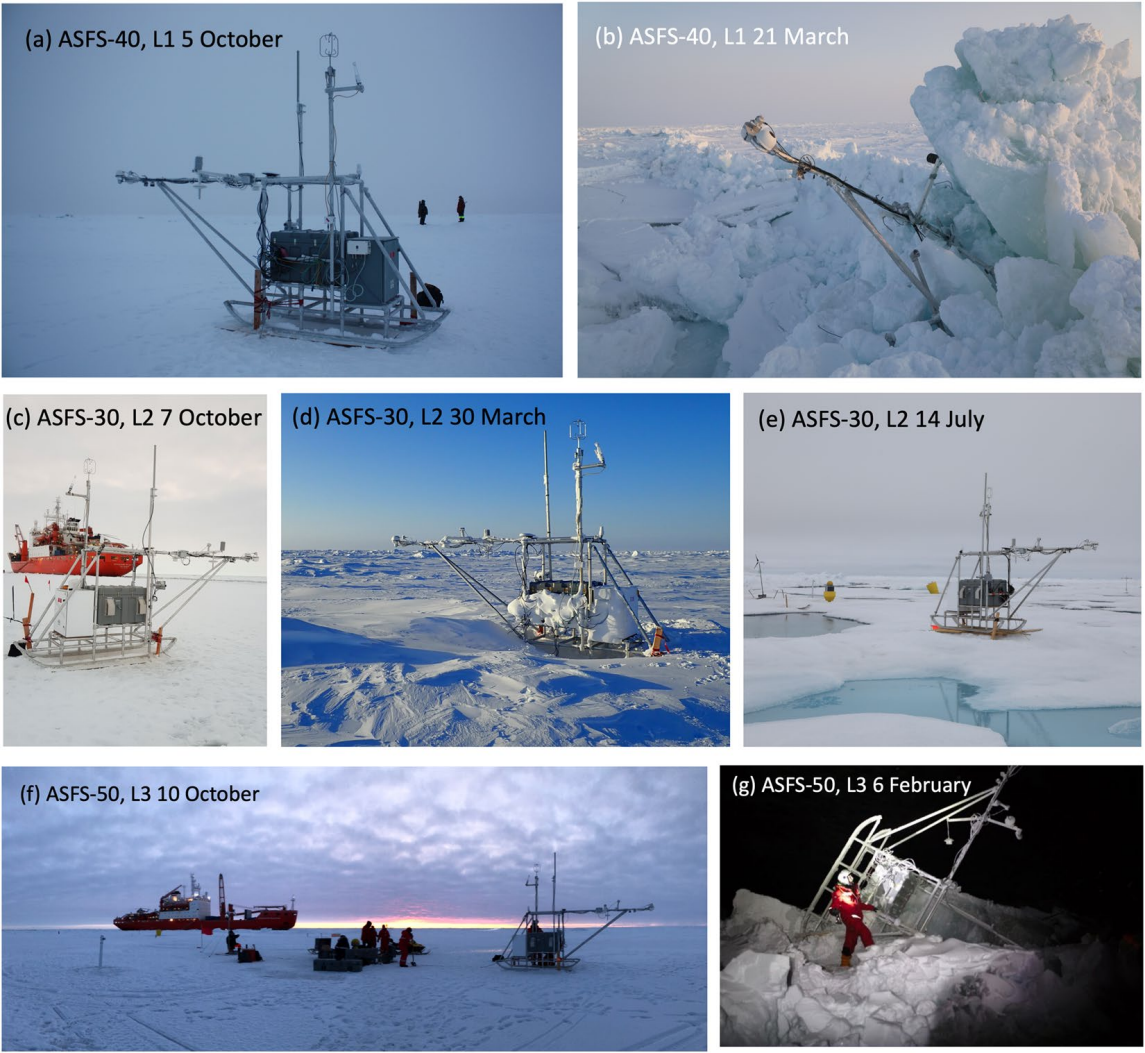
Photos of ASFS in the DN. (**a**) ASFS-40 at L1 on day of deployment, 5 October. (**b**) ASFS-40 at L1 as it was discovered after ridging events of late Feb/early March. (**c**) ASFS-30 at L2 on day of deployment, 7 October, *Akademik Fedorov* in the background. (**d**) ASFS-30 at L2 on 30 March before being removed to the CO and (**e**) on 14 July after it was moved back to L2 after being stationed in the CO from April-June. (**f**) ASFS-50 at L3 on day of deployment, 10 October, *Akademik Fedorov* in the background. (**g**) ASFS-50 at L3 as it was discovered after ridging events of early February.

In early October 2019, an ASFS was installed near an Autonomous Ocean Flux Buoy and an Ice Mass Balance buoy near the floe edge of each L-site. ASFS-40 was deployed at L1 on 5 October on ~1.7 m of ice inclusive of rafted ice layers with saltwater at a depth of 40–50 cm (Fig. 5a). The local snow depth was an average of 9.1 cm based on a series of manual snow depth samples made in an approximately 4 × 8 m area nearby the station. ASFS-30 was deployed at L2 on 7 October on a small, frozen meltpond having a complex profile (8.3/30/30/40 cm snow/ice/water/ice) (Fig. 5c). ASFS-50 was installed at L3 on 9-10 October on a flat pan composed of ~30 cm of ice and 6.5 cm of snow (Fig. 5f). ASFS-40 operated continuously until it went offline on 27 February. The cause of the failure is unknown because it was not possible to reach the site until 17 March when it was found to have been destroyed by a ridge (Fig. 5b). F_LD_ from ASFS-30 is missing prior to 6 November due to a failed fan that caused significant data losses from icing. ASFS-30 was repaired after damage caused by a bear from 8–10 November but otherwise operated continuously until suffering multiple power system failures near the end of March. ASFS-30 was brought to the CO at that time, where it operated until it was returned to L2 at the end of June. ASFS-30 was then brought on board *Polarstern* in late July when L2 was decommissioned (Fig. 5e). ASFS-50 suffered a data gap in late January due to power loss caused by a frozen exhaust line. Similar to ASFS-40, a ridge at L3 severely damaged ASFS-50 on 5 February (Fig. 5g), and it was recovered on 26 March for repair at the CO. Hence, the original scientific design of the atmospheric DN was lost in February 2020, though intermittent, spatial, near-surface atmospheric data on the ~10–15 km scale was obtained from L2 during July 2020.

#### Balloon town & BGC1

Salvage of ASFS-50 in February and the eventual repairs during the first week of April were successful. The sonic anemometer was severely damaged and remained in structurally-poor condition, although operational, having otherwise minimal damage to the majority of ASFS-50 instrumentation. With resupply delayed indefinitely due to COVID-19 and the team’s spare sonic anemometer in Germany being repaired, ASFS-50’s anemometer was field repaired to the extent possible (later meeting both noise and offset specification in 0 wind speed lab tests) and redeployed. For validation, side-by-side comparison data with ASFS-30 were collected in the logistics area at *Polarstern* from 7-14 April.

**Fig. 6:**
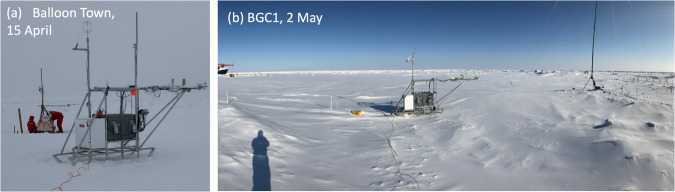
Photos of ASFS at the CO during Leg 3. (**a**) ASFS-30 at Balloon Town on 15 April. The AWI mobile flux sled is in the background. (**b**) ASFS-50 at BGC1 on 2 May. The mast in its third location (13 April – 2 May) is in the background. SIMBA #2020T79^36^ is visible to the left of ASFS-50 in the photo. Note that ASFS-30 was moved into the same position as ASFS-50 in (**b**), replacing it, on 7 May.

In anticipation of a forecasted arrival of warm, moist southerly air masses in mid-April, ASFS-30 was taken to an undisturbed area 35 m south of Balloon Town on 15 April and installed approximately 10 m from a mobile flux sled from the Alfred Wegener Institute (AWI) for coordinated sampling of the event (Fig. 6a). The station had a good field of view to the east, south, and west except for the AWI sled subtending the azimuths of 153–171°. Other CO installations obstructed the field of view from 357-42° (through north). A snow sampling sector containing bamboo flags was to the west. The snow depth at installation was 66 cm. ASFS-30 remained in this location until 2 May when the station was prepared for an extended period of autonomy during the planned departure of *Polarstern* in mid-May for the Legs 3/4 crew exchange.

In anticipation of the possibility of moving Met City due to continuing deterioration of its floe, a second meteorological and SEB site was installed. An area of undisturbed snow having minimal exposure to ice movement was selected near the centre of the CO in the BGC1 sampling sector. The mast was raised there to 23 m on 14 April over snow ranging in depth from 15–40 cm in a slight drift against an old ridge (Fig. 6b). The ice thickness was 400 cm at the guy anchor close to the ridge and ranged from 173–315 cm (mean 256 cm) away from the ridge. ASFS-50 was installed near the mast (Fig. 6b) on 14 April with the boom facing towards Met City on 32 cm of snow. SIMBA^28^ buoy #2020T79^36^ was deployed 2–3 m behind the ASFS-50 on 16 April and the sled was rotated about 90° to optimise the positioning of the anemometer for winds coming from the direction of Met City across sampling sectors BGC1 and Snow 2. The mast was taken down for the final time on 2 May for the planned departure of *Polarstern*. On 7 May, ASFS-50 was also returned to *Polarstern*, and ASFS-30 was moved from Balloon Town into the position previously occupied by ASFS-50 in BGC1, where it would remain while *Polarstern* was away for the Legs 3/4 crew exchange.

#### Met city 2 & first year ice

**Fig. 7:**
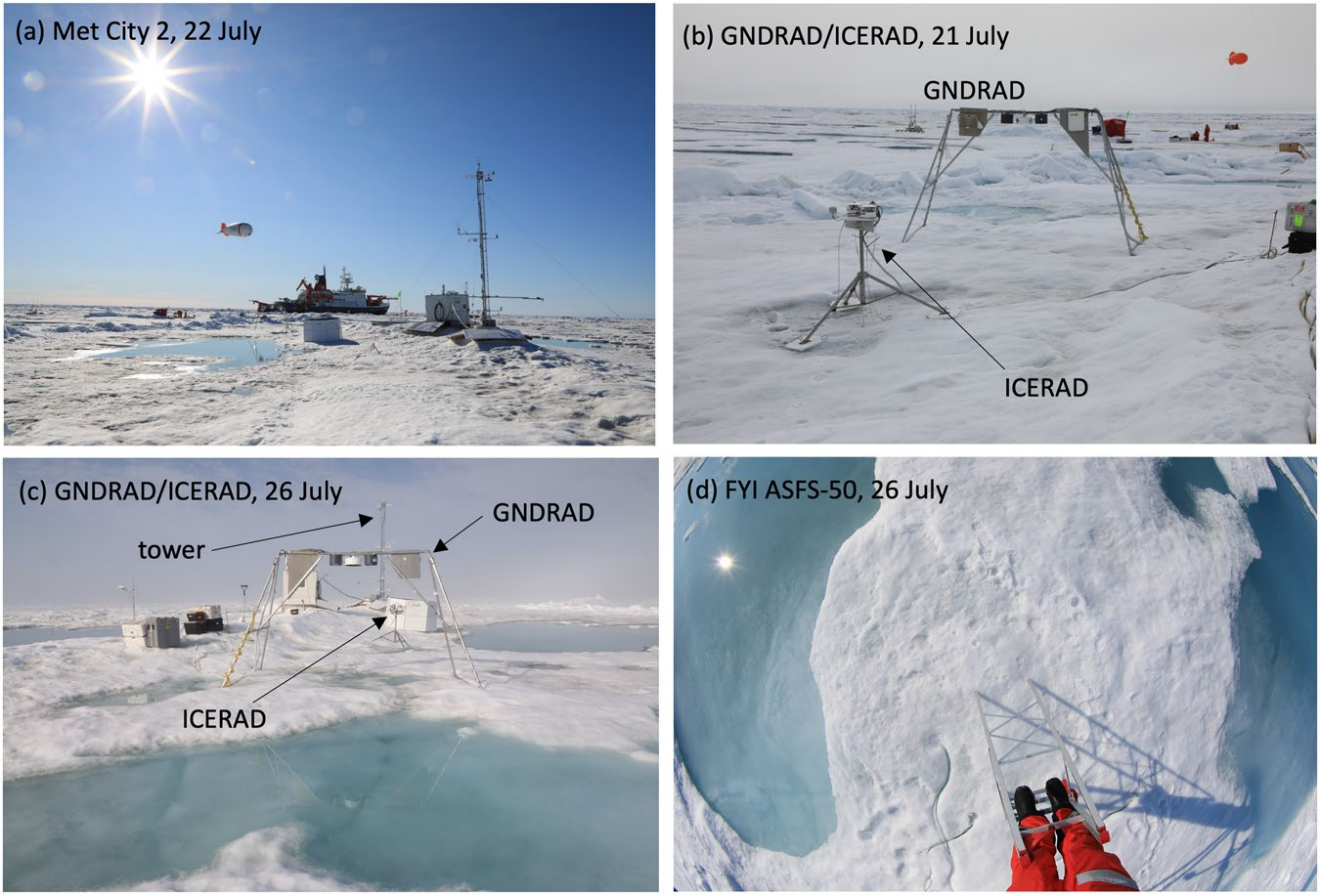
Met City 2 during Leg 4. (**a**) Tower and nearby installations at Met City 2 on 22 July. (**b,c**) ARM ICERAD/GNDRAD systems during period of expansion of melt ponds. (**d**) Wide-angle photograph of the surface beneath the ASFS-50 boom at First Year Ice (FYI) on 26 July.

*Polarstern* returned to the CO in mid-June to discover minimal additional deformation following the 12 May event, but a wet surface from melting snow. ASFS-30 was serviced on 21 June and left to continue measurements while Met City was re-established to maintain continuity between the first and second Met City installations. Many melt ponds were present, but not yet fully defined. A new location for the tower (Fig. 7a) was chosen at 100° and 440 m from the new parking position of *Polarstern* in an area with intersecting ridges that was unlikely to pond. This ridge continued in the direction of *Polarstern* and also beyond Met City along this same general axis. The tower base was ~2 m above the water level (to partially account for this offset, the 2 m sensors were mounted lower on the tower). Anchors for the tower guy lines were drilled through the ice, ~520–540 cm. Measurements at the tower resumed 24 June; the mast was not re-installed.

Most other installations during Leg 4 were positioned between *Polarstern* and the tower. Apart from a sector of about 25° in the direction of *Polarstern*, there were few significant non-natural disturbances in the turbulent field measured by the tower. Measurements from the surface-viewing IRT and flux plates sampled the sloping surface of the ridge. The flux plates were frequently exposed by melting, sometimes day to day, and were often reburied down to the interface with hard ice.

Initially, the ice surface within ~20–30 m of the tower in all directions was not ponded, but there was extensive ponding beyond that in most directions. Because the snow in the ridge was deeper, it took longer to melt than the shallower snowpack over level ice. By late June, meltwater consolidated into ponds, particularly as the snow melted from level areas. During this period, a pond formed near the Met Hut and tower; ablation shielding was installed to minimise melt around the base of the tower and hut (Fig. 7a). Later, the melt ponds expanded and the surface became predominantly bare ice, though with spatial variability in morphology. For example, Fig. 7b,c show surface changes in the vicinity of the albedo measurements during the fourth week of July. Substantial ablation occurred around the Met Hut and tower, which was controlled by shovelling additional snow and ice around the base for insulation.

During Leg 4, ASFS-50 was operated on the part of the CO characterised by first-year ice from 30 June to 30 July, located ~300 m from *Polarstern*. The station was installed in an area of extensive melt ponds (Fig. 7d) and was repositioned slightly (~3 m) on 10 July so the down-looking radiometers viewed a mixture of bare ice and melt ponds in the immediate area. ASFS-30 remained at the CO until 30 June, at which point it was returned to the L2 site with the remaining other autonomous instrumentation.

#### “North pole” CO, met city 3, and hinterland pond

**Fig. 8:**
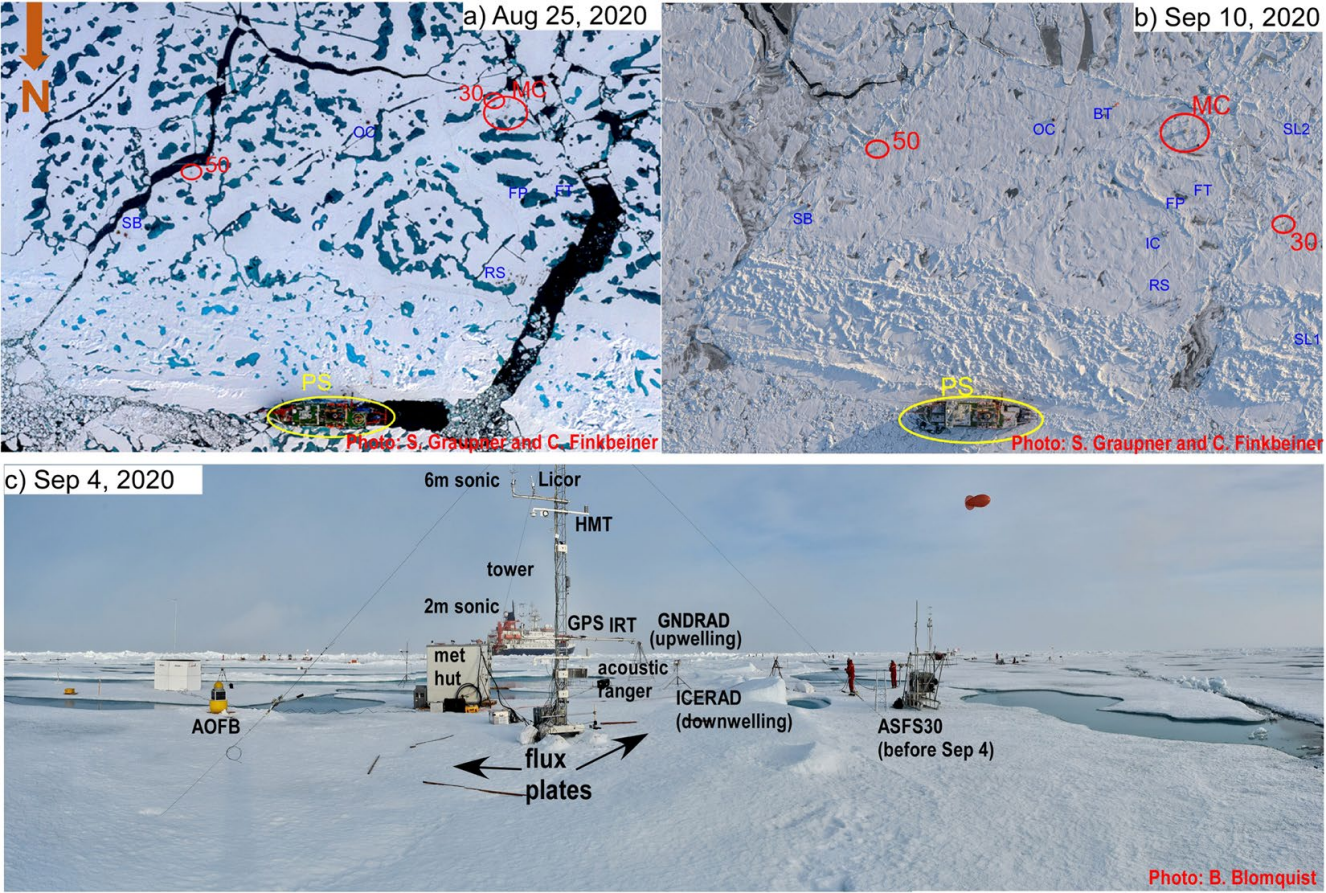
Central observatory floe on (**a**) 25 August and (**b**) 10 September 2020. (**c**) shows Met City 3 instrument installations. The relative locations of Met City 3, ASFS-30 and ASFS-50 are shown in red in (**a,b**), whereas other installations from other teams are shown in blue (SB-submersible; OC-Ocean City; BT-Balloon Town; FT-Fibertown; FP- flux pond; IC – ice coring; RS – remote sensing; SL1, SL2 – AWI EC sleds). The distance between the *Polarstern* (yellow oval) and Met City 3 is ~400 m. Photo credit for (**a,b**) is ©UFA Show&Factual (unpublished, used with permission).

**Fig. 9:**
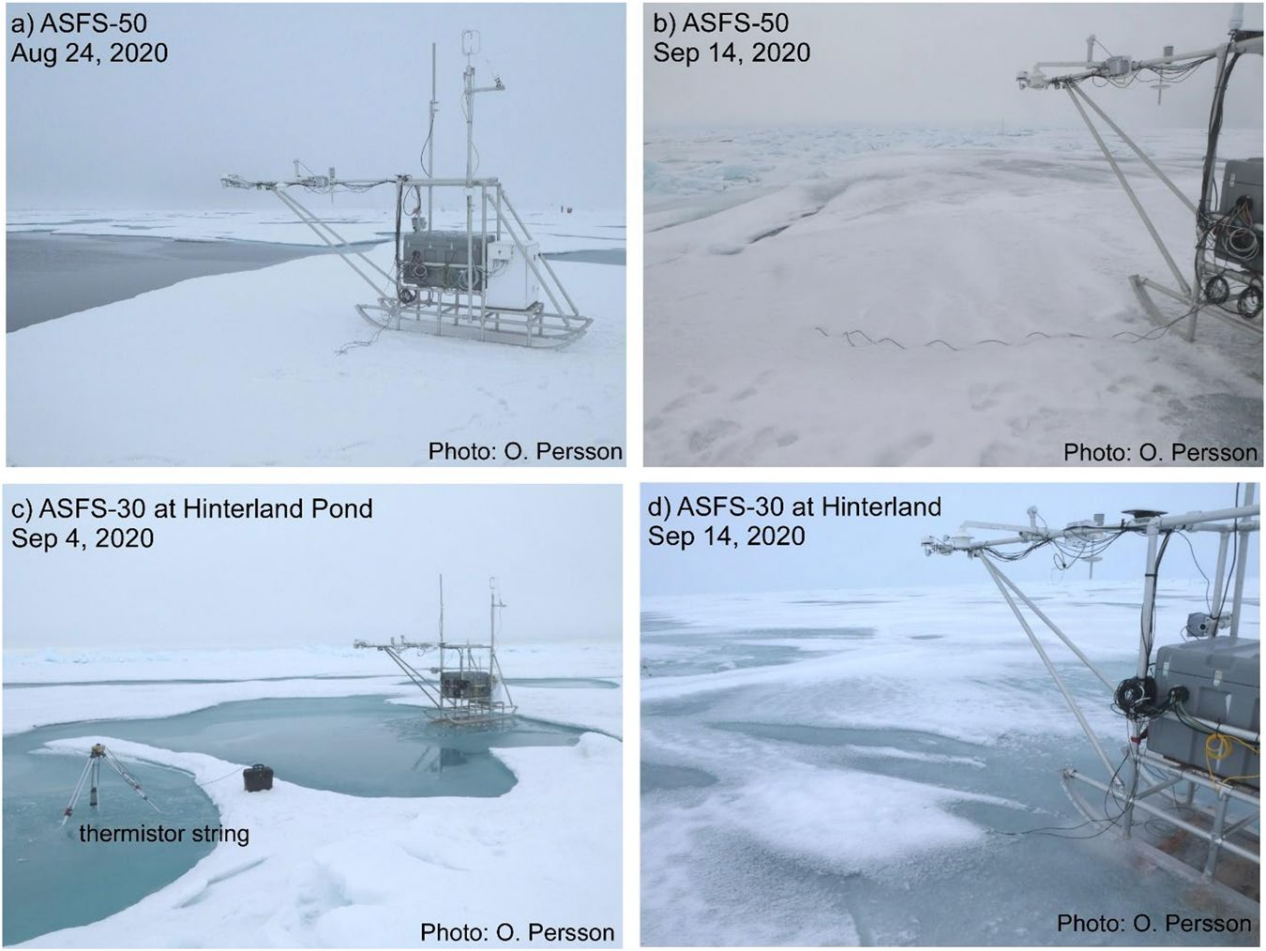
Photos of ASFS at the CO during Leg 5. (**a,b**) ASFS-50 at its location near an active and freezing lead for all of Leg 5; (**b**) ASFS-30 at its location near the met tower from 24 August - 4 September, and (**c,d**) ASFS-30 at its Hinterland location in a melt pond from 4-19 September, capturing freeze-up.

Following the breakup of the original ice floe, *Polarstern* identified a new floe for Leg 5 at 87° 30′ N on 21 August. The floe was ponded with ~1.0–1.5 m thick ice. *Polarstern* moored to the north side of a ~100 m wide, 3–5 m high ridge at the north side of the floe (Fig. 8a,b). Because a primary measurement objective for Leg 5 was to capture the transition from summer melt to autumn freeze-up, which was imminent, ASFS-50 and ASFS-30 were rapidly deployed on 21 August. ASFS-50 was deployed at the southeast edge of the floe near an open lead bounding the floe (Figs. 8a, 9a). ASFS-30 was deployed to the southwest corner of the floe, with the bounding lead, here quite narrow, to its south and west and near a series of meltponds (Fig. 8c). Met City 3 was then built near the ASFS-30 location, with the prone sensor intercomparison begun on 25 August and the tower raised on 27 August. Because the tower was well-established by early September, ASFS-30 was moved on 4 September about 200 m northwest across the lead and placed on pallets in an open meltpond (“Hinterland Pond”) to measure fluxes during the pond freeze-up (Figs. 8b, 9c). A thermistor string was also placed through the bottom of the meltpond into the upper ocean. Although some meltponds in the CO froze faster than others (Fig. 8a,b), Hinterland Pond was open water on 4 September (Fig. 9c). The two AWI flux sleds were also placed in a straight line extending from the first sled within a broad, very rough ridged area, through ASFS-30 at Hinterland Pond, to the second AWI sled on the relatively flat freezing ponded ice (Fig. 8b). The purpose of this deployment was to obtain measurements with which to estimate surface form drag from the ridge.

**Fig. 10:**
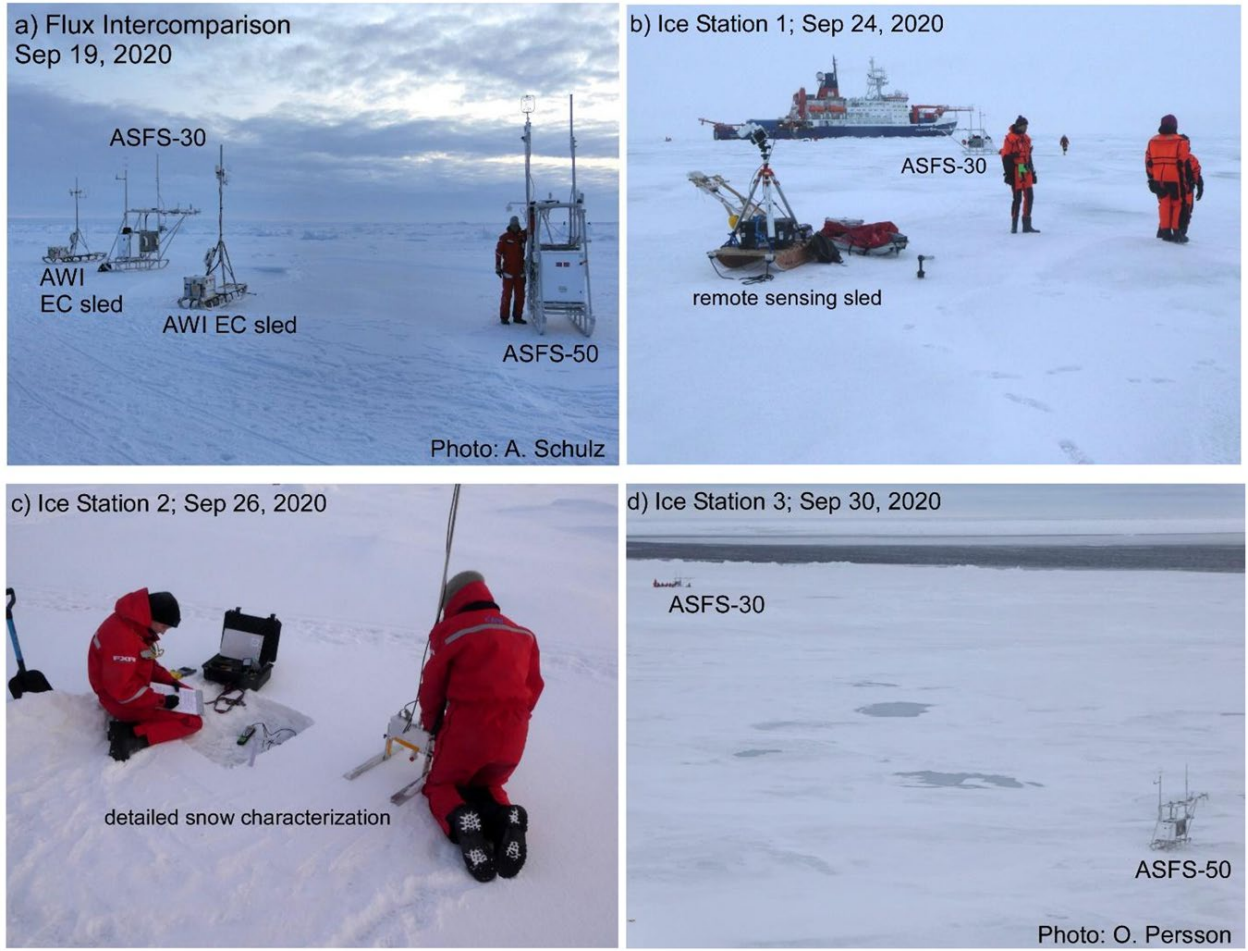
Special measurement periods with the ASFS at the end of Leg 5. (**a**) A turbulent flux intercomparison period was conducted on 19 September between the ASFS and the AWI flux sleds. (**b,c,d**) three ice stations were conducted during the return transit using ASFS along transects to measure the spatial variability of the energy fluxes and obtain independent estimates of emissivity. These were done in conjunction with measurements by the cryospheric and remote sensing teams along identified transects.

Surface conditions varied significantly during Leg 5, but with the trend being towards complete freeze-up. The initial installation on 21 August was on bare ice with a surface layer of large, wet, ice grains, with the ASFS-30 radiometers positioned ~3 m from the edge of a meltpond and those for ASFS-50 ~2 m from the lead (Figs. 8c, 9a). The conductive flux plates at Met City 3 were placed at ~5 cm depth in the ~5–7 cm of snow that had fallen on the morning of 24 August to the southeast (Flux Plate A) and west (Flux Plate B) of the tower. After alternating periods of melt and freeze, final freezing appeared to begin on 4 September. Warmer weather and rainfall occurred with a storm on 14 September, which briefly interrupted freeze-up. The lead bounding the CO floe with Met City 3 was active during the month-long deployment, with intermittent ridging and lead-opening events, as well as freezing (Figs. 8a,b, 9b). On 9 September, a large ice block ~3–4 m tall was raised ~20–30 m to the south of the met tower, and remained there. The images in Figs. 8, 9 illustrate these changing surface conditions. Before departing, both ASFS were moved to near the Remote Sensing installation on 19 September for intercomparison with each other and the two AWI mobile flux sleds used in the Hinterland Pond installation (Fig. 10a).

#### Ice Stations 1, 2, and 3

During the return transit, three ice stations (IS), each about 6 hours in duration, were conducted on 24, 26, and 30 September. The objectives were to (1) use the mobility of the ASFS to obtain measurements of the spatial variability of the SEB to relate to the spatial variability of snow and ice characteristics, especially thickness, and to (2) to obtain measurements in collaboration with the snow and ice team suitable for deriving surface emissivity. ASFS-30 was moved along a transect with several stops of 15–120 min duration along the same track used by the cryospheric and remote sensing teams (Fig. 10b). Some stops were on frozen meltponds ~30 cm thick, others on ice 70–100 cm thick, and others on ~20 cm thick lead ice. Snow depth varied both along each transect and between the three ice stations, with IS-2 having fresh snow 5–8 cm deep (Fig. 10c). ASFS-50 was used for IS-2 and IS-3 at a fixed site to document the temporal variability of the fluxes, allowing a more accurate determination of the spatial variability from the ASFS-30 measurements (Fig. 10d). Figure 10d shows the relative positions of ASFS-30 and ASFS-50 for IS-3 towards the end of the transect done by ASFS-30 between them.

### Data processing

In this section, we describe the principle of the measurements and the post-processing of the data, including calibration, correction, and quality control. Uncertainties are described in Technical Validation. Data were recorded at 1 Hz at the tower and 5 s at the ASFS. Post-processing was generally performed on 1 min averages. The exceptions are that some quality control was performed at 1 Hz for the tower and the sonic anemometer and gas analyser data streams, which were logged at 20 Hz and aggregated as described below. The heights reported in the text are nominal values, but precise installation heights for each measurement are recorded in file attributes and in Table 2. During (re)installation the actual heights were occasionally different and this is also recorded in the attributes along with the time when the change occurred for affected days. The heights of sensors varied further due to the accumulation and drifting of snow, which was measured locally at the tower and ASFS, but not at the mast.

**Table 2:** Actual installation heights (in metres) of meteorological data at the nominal 2/6/10 m positions of the tower and on the ASFS.

Variable	2 m	6 m	10 m	ASFS30	ASFS40	ASFS50
Temperature	1.75 (1.1–2.2)	5.44 (4.8–5.9)	9.34 (8.7–9.8)	2.13 (1.2–2.4)	2.13 (1.9–2.2)	2.13 (1.6–2.4)
Relative Humidity	1.46 (0.8–1.9)	5.24 (4.6–5.7)	9.14 (8.5–9.6)	1.84 (0.9–2.1)	1.84 (1.6–1.9)	1.84 (1.3–2.1)
Pressure	1.65 (1–2.1)	N/A	N/A	1.92 (1.0–2.2)	1.92 (1.7–2.0)	1.92 (1.4–2.2)
Wind/Turbulence	2.66 (2–3.1)	5.68 (5–6.1)	10.54 (9.9–11)	3.86 (3–4.2)	3.86 (3.6–3.9)	3.86 (3.3–4.1)

Note that these are installation heights that changed in time with accumulation and ablation of the surface, which was monitored locally using the sonic ranger. The range of the temporal variability in height is represented in parentheses.

Quality control is tracked in the Level 2 files (see Data Records) using a flagging system. Each measurement variable has a corresponding ordinal flag having five possible states: 0 = good data; 1 = caution; 2 = bad data; 3 = engineering; and −1 = missing. Data classified as bad were determined to be unambiguously so and the data were replaced with a −9999 fill value. Cautionary data indicates there is reason to believe the data might have higher uncertainty than normal or might have been impacted in unquantified ways; e.g., unexpected variability during icing conditions. No differentiation between the reasons prompting a “caution” flag is encapsulated in the files. Engineering data refers to periods when data were collected during testing and are not intended for scientific purposes; these data were also replaced with a −9999 fill value.

#### Basic orientation and geometry

The position of each station was recorded using a Hemisphere v102 dual antenna GPS fixed to the boom of each ASFS and the 2 m boom of the tower. The GPS provided geodetic information and time synching for clocks in the data logging equipment. The data were screened for signal quality (horizontal dilution of precision, signal strength, and number of satellites). Station heading was also recorded for input to the calculation of wind direction and to measure ice rotation. The initial orientations of the station were arbitrary, but the offsets to the azimuthal reference on the anemometers were manually tracked. In addition to random noise at high frequencies, more systematic spurious oscillations were observable in the time series with a period of ~1–3 hours. These features were present during testing at fixed locations on land as well as at MOSAiC and so they are attributed to being artefacts rather than real motions of the ice. The full amplitude was 3–4° and so it was desirable to prevent this from propagating into the calculation of the wind direction. It was not possible to constrain the heading by making use of the larger array of GPS buoys within the area because of ice movement within the array. Instead, the data were filtered using a 6-hour running median, which was found to be suitable for its edge-preserving properties.

From the GPS, several useful geometric variables were calculated and stored in data files for assistance with quality assurance and derivation of high-order metrics. Solar ephemeris calculations were made using the NREL photovoltaic library Python module v 0.8.1^37^, which is based on the calculations of *Reda & Andreas*^38,39^. Two metrics for the zenith angle are provided: the topocentric value and an estimate of the apparent position of the solar disk adjusted for atmospheric refraction with reference near the centre of the visible band and based on meteorology (equation 16.4 in^40^). Additionally, the distance and bearing between the station and the *Polarstern*’s Leica GPS (upper P-deck) were calculated (WGS84 datum).

#### Meteorology

Basic meteorology was recorded at each station. A Vaisala PTU307 was mounted to the lowest level of the tower and to the boom of each ASFS (Fig. 2). A Vaisala HMT337 was mounted to the middle and upper levels of the tower (Figs. 2a, 7a, 8c). For relative humidity, the HMT and PTU both use the same capacitive thin-film polymer sensors, which are heated to prevent frost formation on the probe in the high-humidity environment. Temperature was measured with a Pt100 RTD probe mounted 1 m distant from the humidity probe. These sensors were housed within naturally aspirated radiation shield enclosures. The PTU also features a barometric pressure sensor (capacitive with silicon diaphragm), which is the distinction between it and the HMT. The air pressure observations are with reference to the measurement height (1.65 m) and have not been corrected to sea level. A Vaisala WXT534 was mounted to the top of the mast just below the sonic anemometer featuring a similar humidity and pressure sensor and a Pt1000 temperature probe. Measurements were recorded by digital polling controlled by the data logger.

The capacitance of the thin-film humidity sensor is calibrated to relative humidity (RH); however, because the sensor is heated, the ambient temperature probe is used by the internal electronics to arrive at the ambient RH value, which is reported by the sensor. For humidity metrics derived from the Vaisala sensor package reported values, we use formulations by *Wexler*^41^ consistent with Vaisala (e.g., ref. ^42^). Relative humidity with respect to ice (RHI) is calculated following *Hyland & Wexler*^43^.

Quality control procedures included screening for physically possible limits (PPL) and localised outliers. The data were also scrutinised subjectively. For example, rapid settling of RH for 10–25 min was sometimes observed when stations were first powered on. We interpret this as an equilibration of the heated sensor and affected data were subjectively flagged as bad and removed. Intercomparison data were used to adjust temperature and RH values within the sensor accuracy limits, as described in Technical Validation.

**Fig. 11:**
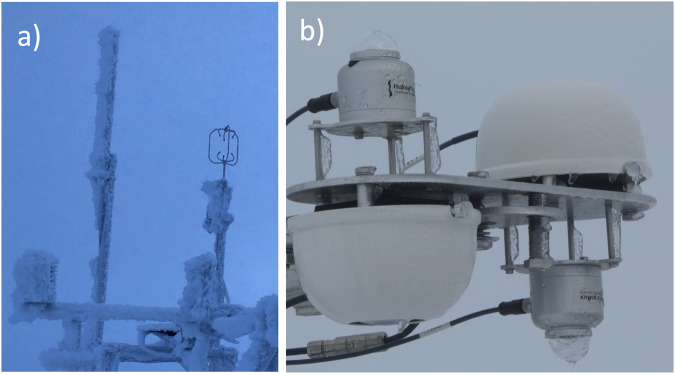
(**a**) Photo of sonic anemometer at L2 on 26 October 2019 during icing conditions being managed on 8 W of heating. (**b**) ASFS-50 3 September 2020 0520 UTC. Rare events of icing on Hukseflux pyranometers occurred on a few days in late August and early September 2020, generally due to freezing fog. These were cleaned on a daily basis.

Metek u-Sonic3 Cage MP ultrasonic anemometers were deployed on the tower and ASFS, and a Metek USA-1 was operated on the mast. Using these, observations of wind velocity in 3 dimensions (x,y,z) were recorded at 20 Hz by each anemometer. The data were used for calculations of turbulent fluxes (described later) and horizontal wind speed and direction. The sonics were mounted nominally at 3.86 m height on the ASFS; at 2, 6, and 10 m on the tower (see Table 2); and at 30 m on the mast (until 18 November and at 23 m height thereafter). Each sonic was heated continuously with 8 or 30 W to prevent the formation of ice on the transducers. The 8 W heating (for ASFS platforms) was found to be sufficient (e.g., Fig. 11a) and there are no known occurrences of data losses at any of the anemometers from icing conditions. Occasionally, dropouts were observed during high winds and attributed to attenuation by blowing snow (sometimes > 1% for sustained winds > 11 ms^−1^ at the 10 m height). The raw data were subjected to PPL and screened for localised outliers to remove anomalous data. Data were rejected if any (of 9) transducer pair paths was reported to be obstructed. Outliers were replaced with the median of values in 10 min segments. The data were then averaged to 10 Hz, and finally rotated from body to earth (u,v,w) coordinate systems using a conventional Eulerian sequence of rotations. Note that “earth” in this case is in fact the inertial reference frame of the sea ice itself. From these components, wind speed and direction were calculated, also in the reference frame of the moving sea ice. Wind direction and the component velocities are reported in meteorological convention. Turbulent flux values were edited for possible impacts from upwind unnatural objects, as described below.

The installation of each sonic anemometer involves visual alignment of the sonic’s intrinsic “north” with the GPS orientation, and the heading from the GPS is then used to calculate the horizontal wind components and the wind direction. The rotational offset between the sonic and the GPS is not known precisely, but is expected to be within 2° at installation. The relative offset between the anemometer on the mast and a GPS located nearby at the surface was also estimated visually and hence made more difficult by the height of the mast. Consequently, the azimuthal orientation of the mast sonic and the wind direction are likely known only to within ~5°.

The tower and ASFS sonics were each equipped with a dual-axis inclinometer at the base of the sensor head that was used for the rotation (the inclination of the sonic on the mast was assumed to be 0 because it did not have an inclinometer). Even after rotating to earth coordinates using the information from the inclinometer, we found a sinusoidal feature that was a function of relative wind direction (+0.81 ± 2.1°), presenting as residual tilt. The residual tilt was found to occasionally change abruptly in time, usually when a system was moved. The source is unknown, though it could perhaps be explained as a misalignment between the inclinometer and the transducers or a systematic bias in the inclinometer reading. The apparent tilt is assumed to be inclusive of u and v, so offsets were found to mathematically level the sensor and were applied as adjustments in the initial set of rotations.

#### Radiative fluxes and surface skin temperature

Each station featured a broadband radiation suite. At Met City, the radiation was observed by the DOE ARM AMF2 “ICERAD” (downwelling radiation) and “GNDRAD” (upwelling radiation) system^44^ positioned within about 20 m from the tower. Both automated^45^ and manual quality control procedures were applied to these measurements. These procedures reviewed the data for signs of icing, sources of measurement instability, errors caused by unlevel sensors, bias, and shadows cast by nearby equipment. The ICERAD was equipped with Eppley PSP and PIR pyrgeometers. Diffuse and spectral solar radiances were also observed, but are not assimilated into data sets presented here (refer to ref. ^34^ for further details). Both the platform and instruments were manually adjusted periodically to keep the sensors level. The upwelling measurements were installed on the GNDRAD structure at a nominal height of 3 m (Figs. 4a,b, 7b,c, 8c). The PSPs and PIRs were calibrated by ARM using the Broadband Outdoor Radiometer Calibration (BORCAL) procedure^46^. All measurements were recorded in analog using Campbell Scientific dataloggers. The PSPs and PIRs were aspirated using DC fans in Eppley ventilators with bevelled radiation shields to better direct air flow. Custom heating coils were installed under the radiation shield to mitigate icing, similar to systems tested by *Cox et al.*^33^.

Each ASFS was outfitted with upward and downward facing Hukseflux SR30-D1 secondary-standard pyranometers (F_SD_, F_SU_) and IR20-T2 pyrgeometers (F_LD_, F_LU_); the former were internally ventilated (1 W) and the latter externally ventilated (7.8 W, Hukseflux VU01). The radiometers were fixed to a common aluminium plate that could be adjusted dynamically using set screws for management of level, but were not gimbaled. The sensor height was ~2 m at a distance of 3 m from the main structure of ASFS. The upward-facing sensors were mounted above the majority of the structure. Adapting the equations from *Bradley & Fairall*^47^ (their Appendix C), we estimate the structure influence in the field of view of the downward-facing sensors to have caused errors <0.1 Wm^−2^ F_LU_ (approximating structure temperature using air temperature) and ~0.3% in albedo (assuming structural albedo of 0.6). Fluxes, instrument level, and fan/heat status indicators were recorded by digital polling of the SR30 sensor. The IR20 measurement was analog. The logger was used to measure the differential voltage of the thermopile and its case thermistor temperature, the latter using a 100 kOhm 0.01% precision reference resistor. All radiative fluxes from the ASFS were derived using the manufacturer calibration. The ventilator fan status was monitored.

Normally, it would be preferred to observe direct and diffuse fluxes independently using a solar tracker to ensure accuracy at low sun angles common in the Arctic. However, this was impractical for an *in situ* sea ice installation and so the primary measurements were global, supplemented by diffuse measurements made by Delta-T SPN1 pyranometers as part of the ARM radiation suite at Met City only^44^. Comparisons between an SR30 and the NOAA Barrow Baseline Surface Radiation Network^48^ (BSRN) tracker at Utqiaġvik, Alaska, using data from* Cox et al.*^33^ agree in the mean to ~1% between 50° and 85° solar zenith angles (not shown), consistent with manufacturer reports of directional response characteristics for the SR30. This assessment provides confidence in the SR30 accuracy even in direct sun at high solar zenith angle. Directional response for PSPs have been studied extensively (e.g., ref. ^49^); the uncertainty calculations found in Technical Validation are inclusive of these errors.

Pyranometers are vulnerable to negative offsets from infrared loss (e.g., ref. ^50^). *Wang et al.*^51^ observed that a negative offset smaller in magnitude than −3 Wm^−2^ in the SR30 becomes a small positive offset (< + 0.75 Wm^−2^) when ventilated. At MOSAiC, ventilation (and usually also heating) was used and nighttime offsets consistent with *Dutton et al.*^50^ were observed. Because of the small magnitudes of the offsets, no corrections are applied. Similarly, observed nighttime offsets for the PSP were deemed to be small so no corrections for infrared-loss^52^ were applied. Although PSPs are known to be vulnerable to infrared-loss, the use of ventilation and heating provides some mitigating thermal control^53^, which likely explains their good performance observed at MOSAiC.

There were a few quality concerns, corrected or flagged in the dataset, that warrant mention here. From 2 December 2019 through 27 January 2020, the ICERAD logger connection from the dome temperature thermistor in the upwelling PIR was loose and gave bad readings. This was corrected by modelling the dome temperature using the case temperature based on an earlier period when both thermistors were functional. This increases the uncertainty in ICERAD F_LU_ (see Technical Validation) by ~0.2–0.3 Wm^−2^ during the affected period (1σ reproducibility was 0.06 C). From 12–21 March, power outages caused the ICERAD downwelling PSP and PIR ventilators to fill with snow, seizing the fans and resulting in a negative bias in the PSP fluxes. The heater and/or fan motors likely warmed the case from below producing an effect similar to infrared-loss. A constant offset (5.2 Wm^−2^) was determined using twilight data and applied to the affected period. The calibration procedure for the PIR is expected to have managed analogous longwave errors. Nevertheless, caution should be exercised in interpreting the downwelling fluxes during this March period near polar sunrise. Some additional quality control flagging was applied to ICERAD based on comparisons with ASFS that does not appear in the ARM archive. Specifically, an unexplained bias in the F_LD_ of ~ + 10 Wm^−2^ was observed relative to nearby ASFS measurements during parts of July and August. The data were scrutinised for potential sources of error but none were confirmed; however, because the discontinuity appeared to be associated with the PIR and it produced unexpectedly warm sky brightness temperatures, the PIR data were flagged as “bad” and removed in the present combined data set. Finally, sometimes the VU01 ventilator fan on the ASFS was switched off to conserve power with the case heater remaining on to provide a buffer against icing. Lab and field tests suggest this led to a + 1.3 Wm^−2^ bias in the unventilated sensor, which we interpret as arising from heterogeneous distribution of the applied heat when unaspirated within the housing. This bias was subtracted off for all times when the fans were off but the heater was turned on.

Melting of the surface in Legs 4 and 5 sometimes caused the level of the ASFS to change by 1–6° over days to weeks. A correction was applied to the F_SD_ measurements following *Long et al.*^54^. A requisite variable for the correction, the pyranometer’s level, was recorded, but not the aspect of the tilt. The aspect was derived by measuring the change in pitch and roll reported by the inclinometer on the anemometer. The anemometer and radiation mount were levelled independently and thus did not share a coordinate frame, though their relative orientation remained fixed between re-levelling. Consequently, it was not possible to correct the level of the radiometer to 0°, but it was possible to correct it back to the level at installation, generally <1°. The correction was only applied to the upward-facing pyranometer and to the proportion of the irradiance originating from the direct beam. Generally, the correction is large when the aspect of the tilt is aligned with the sun’s rays on clear-sky days and is small when the aspect is orthogonal to the sun. The correction factor also approaches unity when the sun is obscured by clouds. To separate the direct and diffuse contributions, ARM’s Delta-T SPN1 was used when it was available. For times when the SPN1 was not available, the diffuse flux was parameterized using data observed in a prior Arctic experiment^33^ for cloudy skies. For clear skies, diffuse flux was estimated as a parameterization of the Rayleigh limit for a snow-covered surface, developed using the STREAMER radiative transfer model^55^ and the Arctic summertime standard atmosphere^56^ in a manner similar to other published parametrizations of the same over low albedo surfaces^45,50,52,57^. The average correction (absolute value) from 26 May – 21 June was 8.4 Wm^−2^. The mean correction when the sky was clear was 34.6 Wm^−2^ and was 1.9 Wm^−2^ under cloudy skies. The 95th percentile was 51.6 Wm^−2^ and the maximum was 92.4 Wm^−2^.

The radiometers used for the ASFS were demonstrated to be effective in mitigating the formation of ice on their domes during a prior experiment in northern Alaska^33^ and were selected for MOSAiC because they also used relatively low power. The SR30 features ~3 W of internal heating and air circulation (+1 W). The IR20 features a small amount of case heating (1.5 W) and was operated within a VU01 ventilator with a 7.8 W fan. The VU01 also features heaters, but these were not used. During MOSAiC, ice was rarely observed on any of the radiometer domes (see also Technical Validation), but identified occurrences of icing, primarily during Leg 5 for downwelling pyranometers, have been flagged in the data set.

Surface skin temperature was derived from the radiative fluxes as follows:

3 $${T}_{s}={\left\{\left[LWU-\left(1-\varepsilon \right)LWD\right]/\epsilon \sigma \right\}}^{0.25},$$

where σ is the Stefan-Boltzmann constant and ε is the emissivity. For snow, ε was set to 0.985, consistent with other studies (e.g., ref. ^13^) and justified by the relatively spectrally flat hemispheric infrared emissivity of snow^58^. For the wintertime snow surface, this assumption is likely within 0.5% (~ 0.04 C at MOSAiC), but during summer when the surface type was variably ice, liquid, or melting snow, the actual emissivity may have differed by more. Intercomparisons amongst *T*_*s*_ derived from pyrgeometers and IRTs during the campaign suggested the IRTs were biased low during cold temperatures. Overall, the pyrgeometers at MOSAiC are thought to have provided the most reliable values across the range of conditions and a “caution” is applied to IRT data when the data was suspect.

#### Conductive fluxes

Several collaborative projects measured temperature profiles through the ice and snow from which conductive fluxes can be derived given assumptions of the thermal conductivity but with the advantage of a full profile (e.g., ref. ^59^). To complement those measurements, we deployed two conductive flux plates at the tower and with each ASFS. The plates measure the conductive flux directly by calibration of the differential voltage of a thermopile set within the plate’s mass, but the measurement is only made at a single layer within the snow profile and is subject to error dependent on differences in the properties between the plate and the matrix^60^ (see Technical Validation). At installation, the flux plates were set into the snow at a depth of 3 cm below the surface (e.g., Fig. 9a), after which accumulation buried them deeper or ablation exposed them and they were reinstalled. Their relative depth is relatable to the change in snow depth, described next. Caution should be exercised in particular during Legs 4 and 5 when they were frequently repositioned and were vulnerable to contact biases due to air pockets and solar heating.

#### Snow depth

Snow depth was observed locally beneath the booms at each ASFS and the tower using Campbell Scientific SR50-A acoustic rangers. The area within the field of view was about 1 m^−2^. These data were screened for PPL and localised outliers, as well as for signal quality, which was reported by the sensor. The reported range was corrected for temperature dependencies on the speed of sound using the colocated Vaisala PTU307. The sensors measured the changing height of the surface with accumulation and ablation. This distance was converted to snow depth by initialising with a manual snow survey carried out during installation. Settling of ASFS and adjustments to the guy lines of the tower caused occasional discontinuities in the SR50 distances. These discontinuities were identified, and the distances were mathematically re-initialized to remove the error.

#### Atmospheric gases

Gas density measurements of water vapour and CO_2_ were recorded at 20 Hz at both the tower and ASFS using Licor 7500-DS open path gas analysers. The uptime for these measurements is more limited than the other data streams because the sensors were generally switched off on the ASFS during the winter to conserve power and were frequently hampered by icing of the upper sensor window at the tower. This was managed by cleanings during daily visits to Met City. In March, a 3 W flexible heater was fitted to the tower sensor, which helped to reduce icing. The 20 Hz data were screened for PPL, localised outliers, and temperature and mechanical stability. The signal of the reference frequency for CO_2_ is sufficiently stable to be used as an indicator for icing: a drop in signal is usually associated with attenuation by an obscured window. Data when the signal strength was below 94% were rejected. Cross-talk with H_2_O at the CO_2_ absorption lines used by the sensor was determined to have a significant effect on fluxes of CO_2_ derived from eddy covariance, and therefore we recommend extreme caution when interpreting the CO_2_ fluxes.

#### Turbulent sensible and latent fluxes

Turbulent sensible (F_s_) and latent (F_l_) heat fluxes were calculated using eddy covariance methodology. The fluxes are reported for the nearest-power-of-2 (po2) integration window at 10 min intervals (13.65 min integration). As with the averages of other variables, the time stamps in the files mark the beginning of the averaging time, but the expansion of the standard 10 min averages by the po2 integration are centred; for example, a 12:00 UTC value for 10 min flux is valid for the averaging period of 12:00-12:10 based on data from 11:58:10 to 12:11:49. The workflow and data preparation are similar to the calculations of F_s_ reported for Arctic coastal stations Eureka (Canada) and Tiksi (Russia) by *Grachev et al.*^32^. The quality-controlled 10 Hz values in earth (u,v,w) coordinates are used as inputs for the flux calculations. The fluxes are computed if at least 50% of the integration window has valid data. First, the earth coordinate frame is rotated into the streamline using a double rotation^61^. Then the power spectral density (Welch) and cross-spectral density are calculated using the Python scipy signal processing module^62^. The FFTs are Hamming filtered and linearly detrended. Spectral smoothing is applied to the covariances, which are then converted to flux in Wm^−2^. The calculation of F_l_ receives a density correction following *Webb et al.*^63^ (3.5% ± 2.8% during autumn and winter). Note that the analogous correction for F_s_, which is very small, was not applied and thus F_s_ formally represents a buoyancy flux. In addition to fluxes, spectra and diagnostic variables are archived as well as stress and momentum terms, including friction velocity of the streamwise component, (${u}_{\ast }=\sqrt{\underline{w{\prime} u{\prime} }}$), the temperature scale (T_*_), the turbulent kinetic energy dissipation rate, the drag coefficient (*C*_*d*_), and the Monin-Obukhov stability parameter (*z*/*L*).

**Fig. 12:**
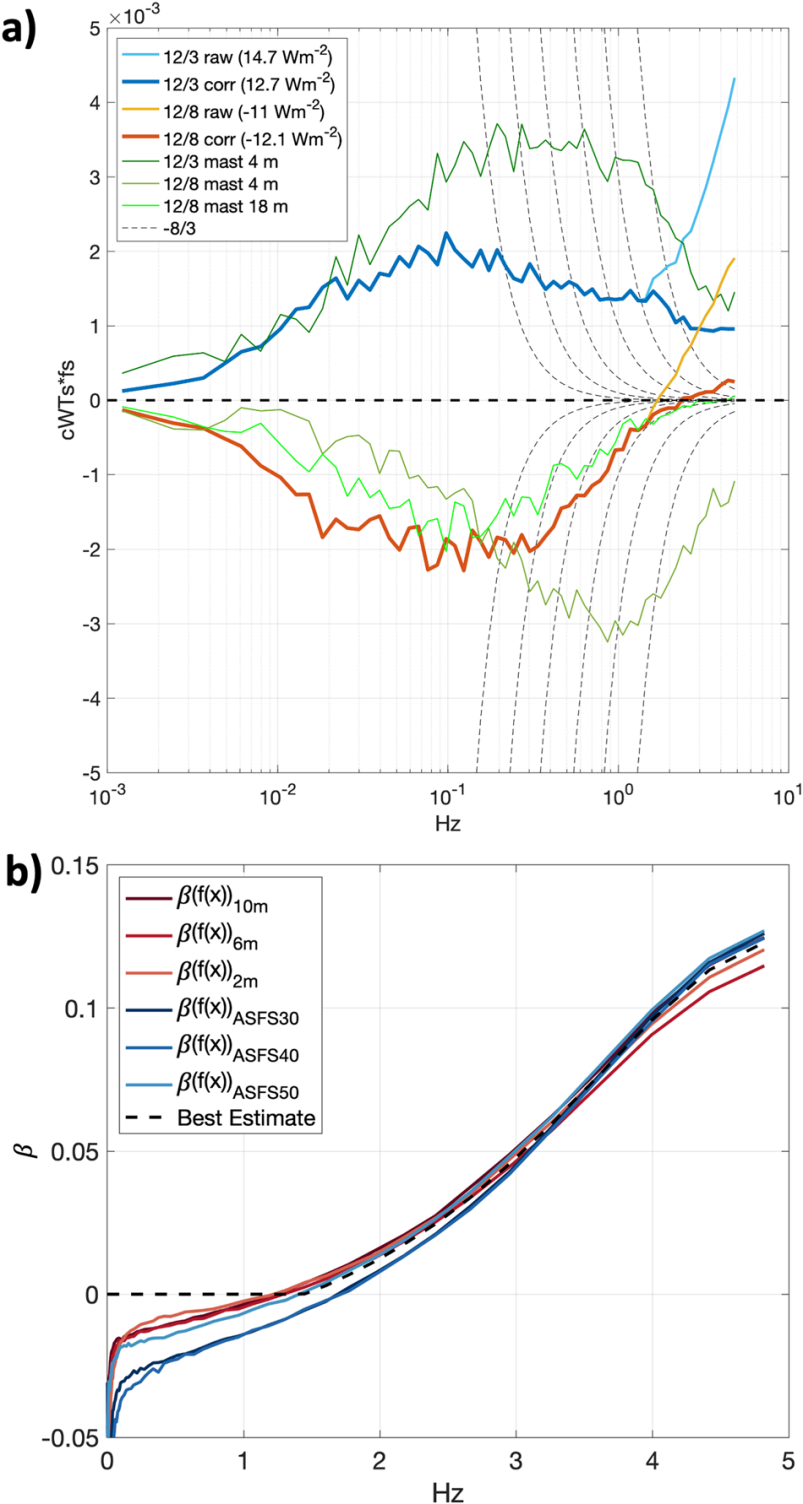
(**a**) Cospectra based on the mean of all sequential 10-minute integration periods from the 10 m tower sensor on two days, 3 and 8 December, 2019, when F_s_ was predominantly positive (cyan/blue) and negative (yellow/red). Cyan and yellow are data prior to the correction while blue and red are after; at frequencies where only the latter is visible, the spectra are identical. Dashed lines are −8/3 reference, here plotted using a linear y-scale. For reference, data from a nearby Metek USA-1 (that did not contain the artefact) that was mounted to the mast is also shown (greens). The mast height was 4 m on 3 December and was raised from 4 m to 18 m on 8 December. The peak being shifted to higher frequencies at the 4 m height is consistent with the sensor being closer to the surface than the 10 m tower sensor. The data after the mast was raised (light green) show the shift toward peaking at lower frequencies, more similar to the 10 m sensor. (**b**) Coefficient, *β*, as a function of frequency under well-mixed (windy and isothermal) conditions. Reds are sensors mounted on the tower and blues are ASFS stations. The number of included times ranged per sensor from N = 3627 (ASFS-40) to N = 7055 (10 m tower).

The cospectra between *w* and *T* (*C*_*WT*_) were found to share non-physical variance at high-frequencies characteristic of noise. While such features are sometimes observable in individual spectra (e.g., ref. ^64^), noise does not typically correlate with *w*, and therefore this error was unexpected. Figure 12a shows examples of daily averaged 10-minute *C*_*WT*_ from two example days observed from the 10 m tower anemometer characterised by persistently positive and negative F_s_. The noise is apparent as a rapid increase toward larger positive values at frequencies >~1 Hz. Because the spectral slopes of the constituents are −5/3 while *C*_*WT*_ is −8/3, the latter drops off more quickly and consequently the error is most apparent at high frequencies. The noise is shared and thus always positive, enhancing otherwise positive F_s_ and suppressing otherwise negative F_s_. The bias was found in all measurements from all uSonic-3 Cage MP model sensors. It is unlikely to be related to transducer heating, being uncorrelated with the amount of heating applied. Similarly, an external noise source (e.g., electrical) is also unlikely: the different station types were powered, grounded, and operated differently. While the error could be related to the cross-wind correction^65^, the unique geometry of the multipath system and a lack of detailed documentation hamper further investigation. However, we were able to remove the shared variance from the 10 Hz data as follows.

We begin by assuming that the error is caused by *T* being contaminated by *w*. Thus,

4 $$T={T}_{ideal}+\beta w,$$

where *β* is a coefficient (described below) and the subscript *ideal* distinguishes idealised from measured values. We determined it was necessary to treat *β* as a function of frequency and to perform the correction in the frequency domain. The measured cospectrum can thus also be thought of as being comprised of individual contributions from the idealised and artefact variance sources:

5 $${C}_{WT}\left(f\right)={C}_{WTideal}\left(f\right)+\beta \left(f\right){S}_{WW}\left(f\right),$$

where *S*_*WW*_ is the variance spectrum of *w* and *f* is the frequency vector. Then, from Eq. (5),

6 $$\beta \left(f\right)=\frac{{C}_{WT}\left(f\right)-{C}_{WTideal}\left(f\right)}{{S}_{WW}\left(f\right)},$$

Clearly, *β* cannot be calculated without *a priori* knowledge of *C*_*WTideal*_. However, *β* can be robustly estimated if the calculation is made using *C*_*WT*_ observed in conditions when *C*_*WTideal*_ is ~0, simplifying the equation. To find *β*, we first composited periods when the boundary layer was isothermal (to meet the aforementioned criterion). In such conditions, *w* is proportional to the horizontal wind speed but because there is no gradient in *T*, there is also no covariance between *T* and *w*. Consequently, F_s_ is ~0 Wm^−2^ and any observable covariance can be interpreted as sampling noise (expected distribution about 0) plus the artefact of leakage of *w* into *T* (not distributed about 0). We used a threshold of 0.1 °C in the absolute value of the gradient from 2 to 10 m height with wind speed > 5 ms^−1^ (to ensure large signal in *w*). Figure 12b confirms that *β* increases with frequency beginning near 1.4 Hz (note that the correlation (r) between *C*_*WT*_ and *S*_*WW*_ also increases from ~0.36 < 1 Hz to ~0.88 > 1 Hz). Lower frequencies are more prone to under-sampling and non-stationarity and therefore poorly constrained. The figure shows results from all three sensors on the tower (2 m, 6 m, and 10 m), as well as the three ASFS stations, demonstrating consistency amongst instruments. The distribution of *β* (representatively taken at 3 Hz) has *μ* = 0.0511, σ = 0.0251, and is not substantially skewed (skewness = −0.6). There is a slight tendency for *β* to increase with temperature but this is a small effect that can be ignored. To construct *β*(*f*), we averaged the results from the sensors and chose 1.4 Hz as a cut-off frequency where lower frequencies are assigned *β* = 0. Finally, we restate Eq. (4) with the modification that the scaling is performed in the frequency domain thusly,

7 $${T}_{ideal}=T-{{\mathcal{F}}}^{-1}\left\{\beta \left(f\right){\mathcal{F}}\left\{w\left(f\right)\right\}\right\},$$

where $\boldsymbol{\mathcal{F}}$ and $\boldsymbol{\mathcal{F}}$^−1^ indicate the forward and inverse Fourier Transform. All covariance calculations were then performed using *T*_*ideal*_ from Eq. (7). Results are shown in Fig. 12a. The daily mean of the error that was removed for the two cases are +19% and +12%. Alternative methods for estimating the magnitude of the error by interpolation across the artefact^64,66^ yielded corroborating estimates for the magnitude of the bias (not shown).

**Fig. 13:**
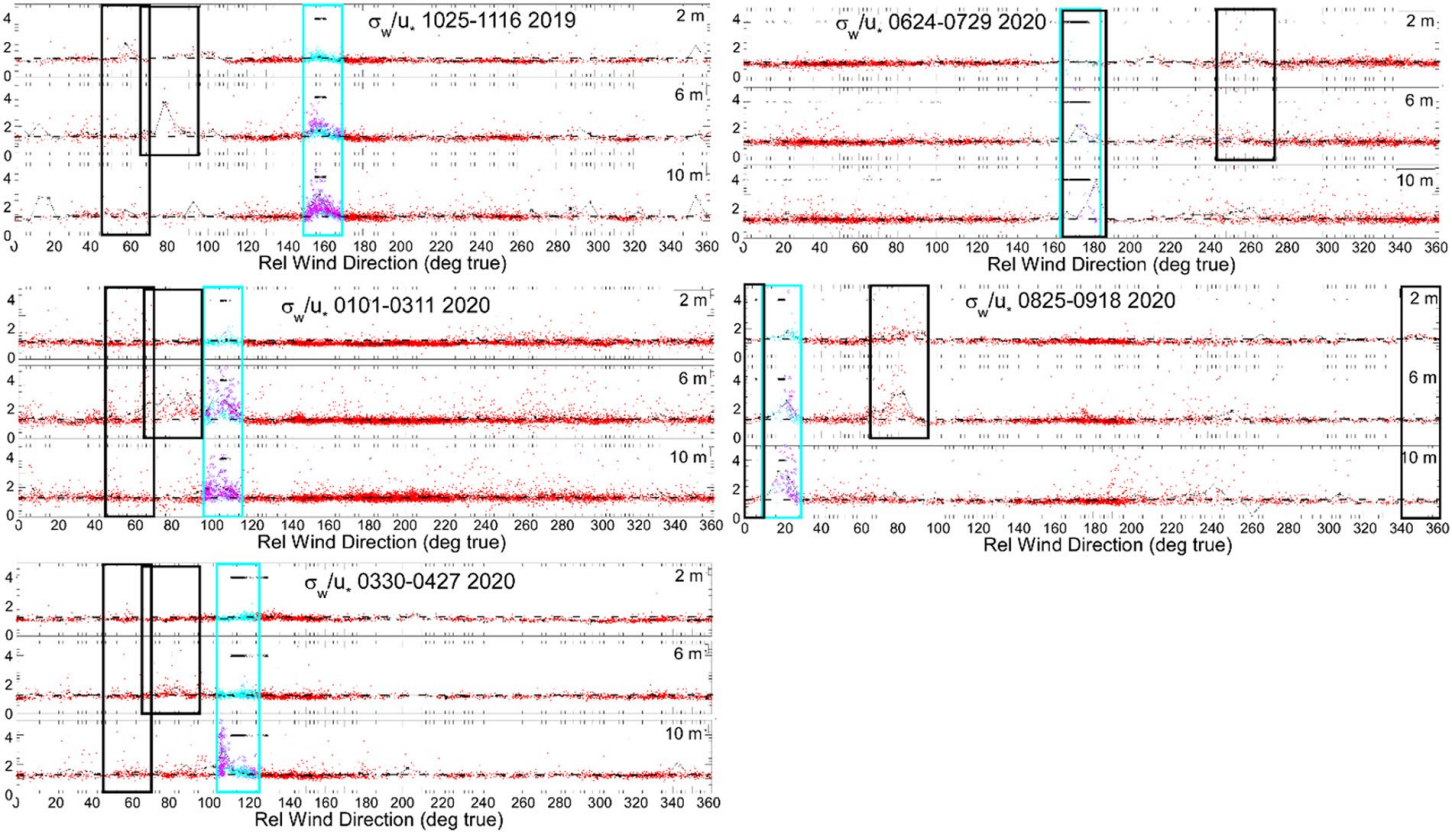
Scatterplots of *σ*_w_/u_*_ (in 10 min segments) as a function of tower-relative wind direction for five time periods during the MOSAiC year. The horizontal black dashed line is the expected value of 1.25. For each period, the boxes show the points for airflow from the ship (cyan), the tower (black; 2 m and 6 m only), and the Met Hut (black; all levels) towards the respective sonic anemometers. The points for which the airflow is coming from the ship (cyan) and the distance to the ship is less than the calculated turbulent footprint size (see text) are circled in magenta. Values when airflow is from the ship are often elevated, especially for the 10 m height when the ship distance is less than the footprint size. Values for winds from the tower and the met hut are often elevated, but not as consistently and as significantly as from the ship at 10 m height.

Flagging for the turbulent fluxes includes PPL and manual editing. These flags are applicable to all eddy covariance calculations, documented in a single flag variable “turbulence_qc”. We have not explicitly flagged data that could be affected by non-stationarity, large angles of attack, or unrealistic spectral slopes, for which the thresholds are highly subjective. Diagnostics relevant to these sources of potential error are available in the Level 2 files for interested researchers. The turbulence data have also been flagged for possible impacts from identified man-made structures upwind of the stations and documented in a flag variable “wind_sector_qc_info” that encodes also the feature responsible for influencing the data. Due to a relative dominance of natural roughness features, scientific infrastructure and human activities in the area appear not to significantly alter the turbulent fluxes measured by the tower or mast, except for the tower frame itself, the Met Hut structure that serviced the tower, and *Polarstern*. This is illustrated by Fig. 13, which shows a useful metric, σ_w_/u_*_, as a function of the tower-relative wind direction, where *σ*_w_ is the standard deviation of the vertical wind component. Monin-Obukhov Similarity Theory predicts this value should be ~1.25 (ref. ^67^). The metric increases if the vertical velocity variance is large relative to its covariance with u, and it is therefore useful for identifying wind directions affected by unrepresentative features. The relative direction of the *Polarstern* changed as the ship moved relative to the tower because of ice dynamics events throughout the field program, though this direction and distance were measured. For Legs 1–3, the tower-relative wind direction to the tower and the Met Hut did not change, though it did change with the different Met City configurations for Legs 4 and 5. All of the 10-min periods for which the airflow came from one of these three structures have been identified, as in Fig. 13. Furthermore, the data points with airflow from the ship were deemed to be “bad” and removed if the ship was within the footprint distance estimate, *L*_*c*_, given by

8 $${L}_{C} \sim \frac{1}{4}\frac{{Z}_{b}}{\left[2{\left(\frac{u* }{U\left({Z}_{b}\right)}\right)}^{2}\right]},$$

which is derived from the concepts given by *Mason*^68^. In Eq. (8), modified from Mason’s^68^ equation (10), *Z*_*b*_ is the sonic measurement height, u_*_ is the friction velocity, and *U* is the wind speed. The 1/4 scale factor was estimated through empirical means as a threshold separating outliers in σ_w_/u_*_. These “bad” points are given by the magenta circles in Fig. 13, which shows that these points have much higher values of σ_w_/u_*_. Additionally, data points from the direction of the Met Hut or the tower that had small or large values, σ_w_/u_* < _0.8 or σ_w_/u_* > _1.8, are also removed. All remaining data points from the direction sectors impacted by either the ship, the Met hut or the met tower are given a “caution” flag. This approach was further validated by comparing to more sophisticated footprint calculations^69^ for a subset of cases (not shown). For ASFS, because the stations were usually deployed distant from significant artificial features, the wind sector flagging was only applied in certain circumstances and those sectors were identified by observation in the field.

**Fig. 14:**
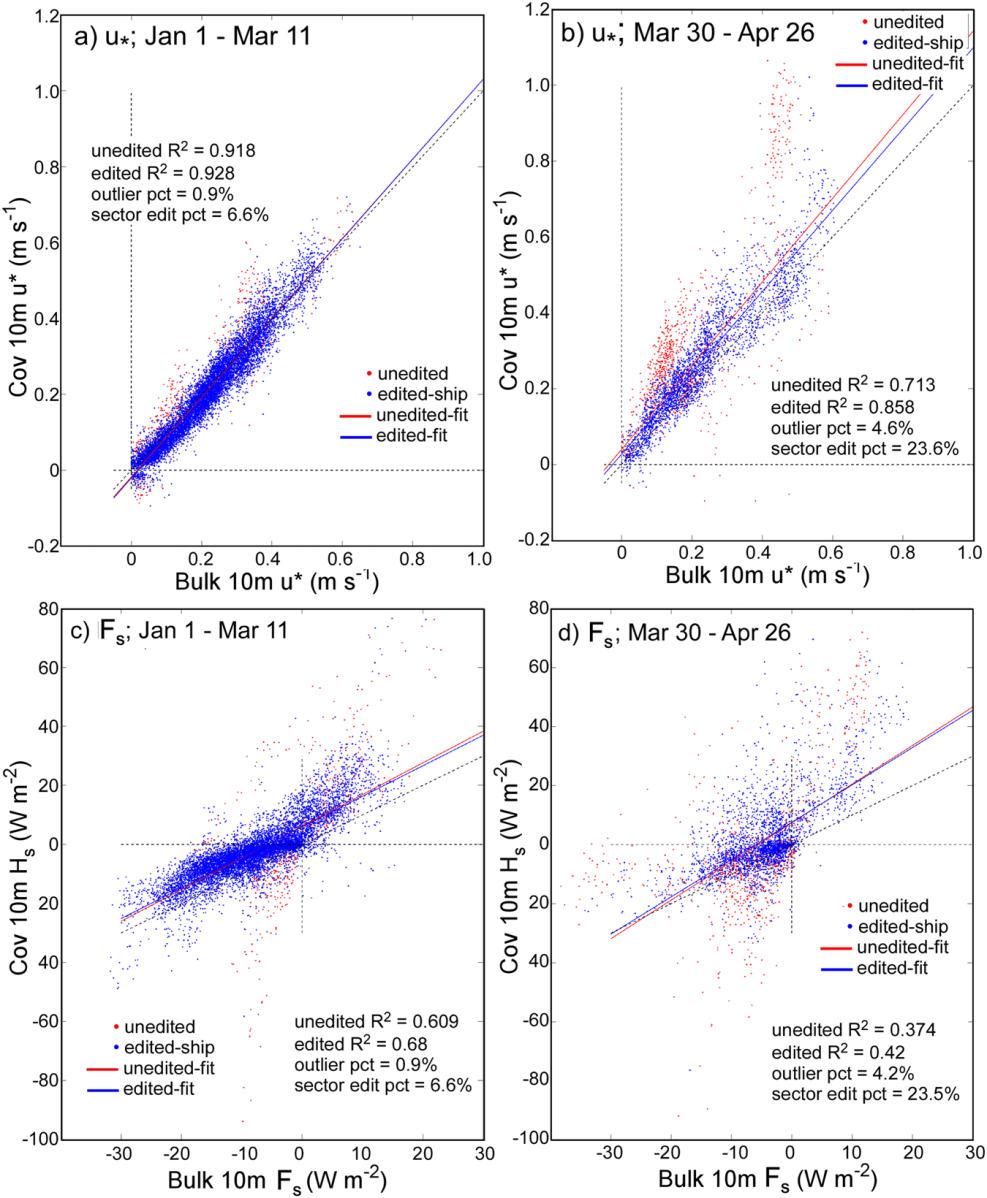
Impact of editing eddy covariance values when plotted as a function of bulk values for two MOSAiC time periods. Shown are (**a,b**) u_*_ and (**c,d**) F_s_. The red points are the ones removed by the editing routines. Also shown are the correlation coefficients before and after editing and the percentage of points removed. The red and blue lines show the best fit to the unedited and edited data points, respectively. The black dashed line is the 1:1 line. See text for further information.

The impacts of the editing of the turbulent flux data described above were examined for the five time periods shown in Fig. 13. The average percentage of points removed was 10.5%, with a significant variation throughout the year from 0.6% (24 June - 29 July) to 23.6% (30 March - 26 April). The editing described above had a positive impact on the data set as shown by the comparison of the eddy covariance u_*_ and F_s_ with its bulk counterpart. The removed data points were primarily located on the fringes of the point clusters (Fig. 14), and the correlation coefficients increased for every time period and for both parameters. Figure 14 also shows the low bias of the bulk fluxes mentioned previously.

To complement the eddy covariance fluxes, and to facilitate gap-filling, F_s_, F_l_, and u_*_ (as well as related stress variables) were also calculated for 10 min averaging periods using bulk methodology. Briefly, the fluxes are proportional to the vertical gradient in temperature (F_s_) and humidity (F_l_) between the surface and a reference height by transfer coefficients that are derived from Monin-Obhukov similarity theory. Because the Obhukov length (*L*) is dependent on the transfer coefficients as well as the input meteorological variables, in practice the algorithm operates iteratively to converge the interdependent equations^70,71^. The algorithm used here^72,73^ is similar to methods developed for open ocean^70,71^, but is tuned for compact, snow-covered sea ice and stable stratification of the wintertime polar oceans. Because of the emergence of spatial heterogeneity in summer inclusive of ice melt and snow, open water, and melt ponds, the values are less robustly constrained. During winter the effect of leads is not captured by the bulk fluxes and in the presence of vapour plumes from leads, the results may be of the wrong sign because the algorithm is bounded by the skin temperature of the snow rather than the open water. Furthermore, the algorithm varies the fluxes with stability, but not with the roughness length. Roughness lengths for temperature (z_T_) and wind (z_0_) are set to 1e-4 and 3.27e-4 m following results at SHEBA^74^; however, the actual roughness of the surface at MOSAiC, in particular in spring, was likely larger due to more frequent and larger ridging. During affected times when an instrument was downwind of ridges, the calculated bulk fluxes are likely too small. We consider the nominal calculation of the bulk flux to be a useful product for interested researchers and thus we have provided these data in the files, whereas the caveats listed here present intriguing basic research problems to address in future detailed scientific studies.

## Data Records

Processed data are archived in daily files in a self-documenting annotated NetCDF4 format. Files may be downloaded from the National Science Foundation’s Arctic Data Center (ADC, https://www.arcticdata.io) under the Creative Commons Universal 1.0 Public Domain licensing. Within the ADC archive structure, the data are distributed with taxonomic grouping by both level and station, with each branch assigned a unique DOI. Within each grouping, multiple file types may be found. The Level 1 files^75–78^ contain the raw data reorganised from their native formats for convenience into ingest files with only technical corrections (e.g., sign convention, variable name) applied. Separate files for each day are made containing 1 minute means (“slow”) and 20 Hz samples (“fast”). These Level 1 data are made available, but are not recommended for general use. For most applications, the processed and quality-controlled Level 2^79–82^ and Level 3^83–86^ files should be used. There are three separate file types for each day for Level 2: 1 minute means (“met”); 10 Hz means (“10hz”); and 10-minute means, also containing turbulence variables (“seb”). Level 3 data are similar to Level 2 but packaged in a more convenient form for users. Level 3 is inclusive only of 10 min means of scientifically-relevant variables (i.e., no diagnostics), and all data not meeting the quality-control flags = 0 or 1 (good or caution data) are removed. Codes for all flags are defined in each file’s global attributes. Table 3 summarises the files and their associated DOIs. Level 3 files are recommended for most applications, while Level 2 files might be recommended for users with a strong background in turbulence and interested in detailed turbulence diagnostic information.

**Table 3:** Data set access information.

Name	Level	Content	format	ADC DOI
10 m meteorological flux tower measurements	1 (raw)	1 min means	mosflxtowerslow_level1_YYYYMMDD_hhmmss.nc	10.18739/A2VM42Z5F
10 m meteorological flux tower measurements	1 (raw)	20 Hz samples	mosflxtowerfast_level1_YYYYMMDD_hhmmss.nc	10.18739/A2VM42Z5F
ASFS 50 measurements	1 (raw)	1 min means	moasfs50slow_level1_YYYYMMDD_hhmmss.nc	10.18739/A2445HD46
ASFS 50 measurements	1 (raw)	20 Hz samples	moasfs50fast_level1_YYYYMMDD_hhmmss.nc	10.18739/A2445HD46
ASFS 40 measurements	1 (raw)	1 min means	moasfs40slow_level1_YYYYMMDD_hhmmss.nc	10.18739/A2CJ87M7G
ASFS 40 measurements	1 (raw)	20 Hz samples	moasfs40fast_level1_YYYYMMDD_hhmmss.nc	10.18739/A2CJ87M7G
ASFS 30 measurements	1 (raw)	1 min means	moasfs30slow_level1_YYYYMMDD_hhmmss.nc	10.18739/A20C4SM1J
ASFS 30 measurements	1 (raw)	20 Hz samples	moasfs30fast_level1_YYYYMMDD_hhmmss.nc	10.18739/A20C4SM1J
10 m meteorological flux tower measurements	2 (processed)	1 min means	mosmet_metcity_level2_1min_YYYYMMDD_hhmmss.nc	10.18739/A2TM7227K
10 m meteorological flux tower measurements	2 (processed)	10 Hz samples	moswind10hz_metcity_level2_YYYYMMDD_hhmmss.nc	10.18739/A2TM7227K
10 m meteorological flux tower measurements	2 (processed)	10 min means & turbulence	mosseb_metcity_level2_YYYYMMDD.hhmmss.nc	10.18739/A2TM7227K
ASFS 50 measurements	2 (processed)	1 min means	mosmet_asfs50_level2_1min_YYYYMMDD_hhmmss.nc	10.18739/A2251FM5R
ASFS 50 measurements	2 (processed)	10 Hz samples	moswind10hz_asfs50_level2_YYYYMMDD_hhmmss.nc	10.18739/A2251FM5R
ASFS 50 measurements	2 (processed)	10 min means & turbulence	mosseb_asfs50_level2_YYYYMMDD.hhmmss.nc	10.18739/A2251FM5R
ASFS 40 measurements	2 (processed)	1 min means	mosmet_asfs40_level2_1min_YYYYMMDD_hhmmss.nc	10.18739/A29P2W74F
ASFS 40 measurements	2 (processed)	10 Hz samples	moswind10hz_asfs40_level2_YYYYMMDD_hhmmss.nc	10.18739/A29P2W74F
ASFS 40 measurements	2 (processed)	10 min means & turbulence	mosseb_asfs40_level2_YYYYMMDD.hhmmss.nc	10.18739/A29P2W74F
ASFS 30 measurements	2 (processed)	1 min means	mosmet_asfs30_level2_1min_YYYYMMDD_hhmmss.nc	10.18739/A2K649V1F
ASFS 30 measurements	2 (processed)	10 Hz samples	moswind10hz_asfs30_level2_YYYYMMDD_hhmmss.nc	10.18739/A2K649V1F
ASFS 30 measurements	2 (processed)	10 min means & turbulence	mosseb_asfs30_level2_YYYYMMDD.hhmmss.nc	10.18739/A2K649V1F
10 m meteorological flux tower measurements	3 (final)	10 min means & turbulence	mosseb_metcity_level3_YYYYMMDD.hhmmss.nc	10.18739/A2PV6B83F
ASFS 50 measurements	3 (final)	10 min means & turbulence	mosseb_asfs50_level3_YYYYMMDD.hhmmss.nc	10.18739/A2XD0R00S
ASFS 40 measurements	3 (final)	10 min means & turbulence	mosseb_asfs40_level3_YYYYMMDD.hhmmss.nc	10.18739/A25X25F0P
ASFS 30 measurements	3 (final)	10 min means & turbulence	mosseb_asfs30_level3_YYYYMMDD.hhmmss.nc	10.18739/A2FF3M18K

## Technical Validation

Table 1 summarises the uncertainties of the measured variables. Broadly, we define uncertainty as the calibration uncertainty of the sensor in accordance with the manufacturer, unless more information is available, in which case the total uncertainty is reported as the combination of all sources in quadrature. We do not distinguish here between sources that result in random and bias errors. There are several variables where more detail is warranted, described next.

### Radiation

A significant source of instantaneous bias in radiative fluxes in the Arctic is the build-up of ice on sensor domes. Using data collected in northern Alaska in 2017–2018, *Cox et al.*^33^ analysed such biases and reported on the ice mitigation performance of the radiometer models having similar configurations of ventilation and heating used at MOSAiC. The IR20, SR30, PSP, and PIR were found to mitigate ice formation on their domes ~99%, ~90%, 70–80%, and ~99% of the time icing conditions were present. Reports from technicians at MOSAiC suggest these performance levels were likely met, or in particular for the case of the PSP, exceeded. Icing was reported during several cases of freezing drizzle/fog that occurred on 28 April, during heavy riming on several days in September (Fig. 11b), and when the fan failed on one VU01 October 2019. These cases were flagged in the data set. Icing is not considered a significant source of uncertainty at MOSAiC owing to the performance of these instruments and platforms.

**Fig. 15:**
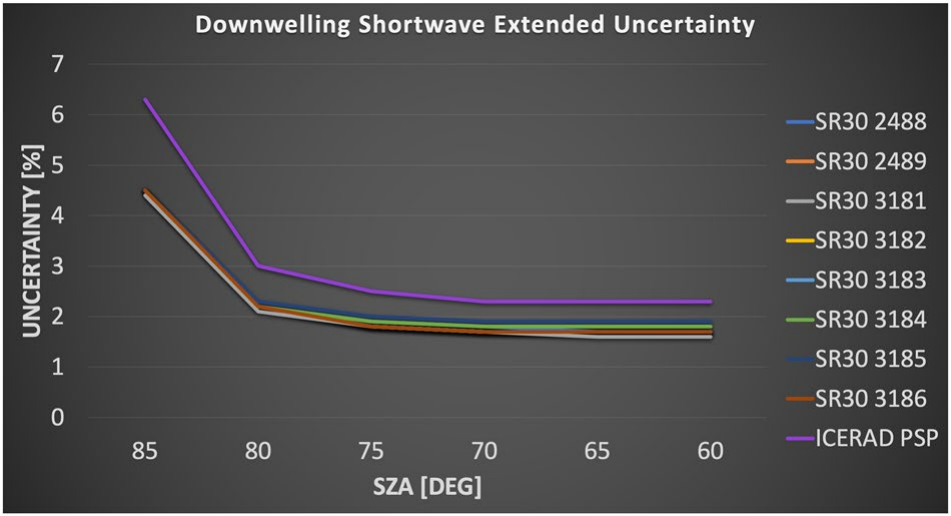
Estimates of uncertainty in F_SD_ as a function of solar zenith angles (SZA) representative of the range observed at MOSAiC. Calculations are valid for clear skies. Diffuse fluxes are estimated to have a flat uncertainty of 3 Wm^−2^. Calculations are made individually for each SR30 using unique values provided by the manufacturer, temperature response, and directional response. All other values, including all values for the PSP are nominal specifications of the instrument model.

*Cox et al.*^33^ also performed an intercomparison of a series of radiometers of various makes and models, including the SR30 and IR20. These operational Arctic repeatability calculations were based on ice-free measurements and were found to be consistent with targets set by the BSRN and other studies^87,88^. Though not formal uncertainty analyses, they may be considered a useful guide for uncertainty, including at MOSAiC. For F_SD_, *Cox et al.*^33^ found repeatability of ~2% for solar zenith angles <80°, increasing for higher angles. The uncertainty in F_LU_ and F_LD_ under cloudy skies was ~ 1 Wm^−2^ whereas under clear skies, the uncertainty increases to ~2–4 Wm^−2^; the average over all conditions was 2.6 Wm^−2^. Values for F_LD_ in Table 1 are taken from *Cox et al.*^33^ and for F_LU_ are assumed consistent with the values found for F_LD_ under cloudy skies. A more formal estimate of uncertainty in accordance with ISO 98-3 standards^89,90^ was calculated for the solar fluxes at MOSAiC, incorporating the following sources: calibration, non-linearity, zero-offsets types a and b (related to temperature gradients in the instrument), non-stability (drift), non-linearity, directional response (related to incident angle), temperature dependency, and level. The results are shown in Fig. 15 and are valid for F_SD_ under clear-skies. To estimate an uncertainty for diffuse F_SD_, the calculation was repeated using only sources unrelated to the geometry of incident radiation at three values of F_SD_ representative of the diffuse fluxes observed during MOSAiC, 150, 200, and 250 Wm^−2^. Results yielded a mean uncertainty of 3.1 Wm^−2^ (ASFS) and 4.5 Wm^−2^ (ICERAD) with weak dependency of F_SD_. These diffuse values are also reported in Table 1 as approximations for F_SU_, but note that variability in F_SU_ is also affected by surface topography and geometry of the incident radiation, in particular in the presence of anisotropic scattering (e.g., sheet ice).

### Conductive fluxes

No corrections were made to the flux plates, which - in addition to intractable sources of uncertainty such as level, solar heating, and contact errors - are subject to deflection error caused by differences in the conductivity of the plate relative to the matrix^60^ and resistance error caused by the plate changing the total resistance of the medium. Resistance error is assumed negligible. The conductivity of the flux plate is ~0.76 Wm^−1^K^−1^ (manufacturer spec) whereas snow over sea ice ranges from 0.087–0.528 Wm^−1^K^−1^ (ref. ^91^). We combine an estimate of deflection error following *Sauer et al.*^60^ with ISO 98-3 manufacturer stated sources (non-stability, temperature dependence, calibration, voltage logging) to find a range of total uncertainty of 7–14%, dependent upon the conductivity of the snow medium. Under certain conditions, in particular during the summer melt period, biases may be significantly larger and caution should be exercised. Note that the relationship between the flux observed by the plate and F_ct_ (Eq. 1) is dependent on the depth of the plate within the snow, with the energy transfer through the layer between the plate and the surface increasing the thickness over which the calculation is balanced by a non-negligible amount, and resulting in the need to incorporate a storage term into the calculation (e.g., ref. ^30^). The determination of the storage term is dependent on the desired parameters of the SEB calculation and varies with the layer thickness resulting from the depth of the plate and therefore we have not provided these calculations.

### Air Temperature and humidity

**Fig. 16:**
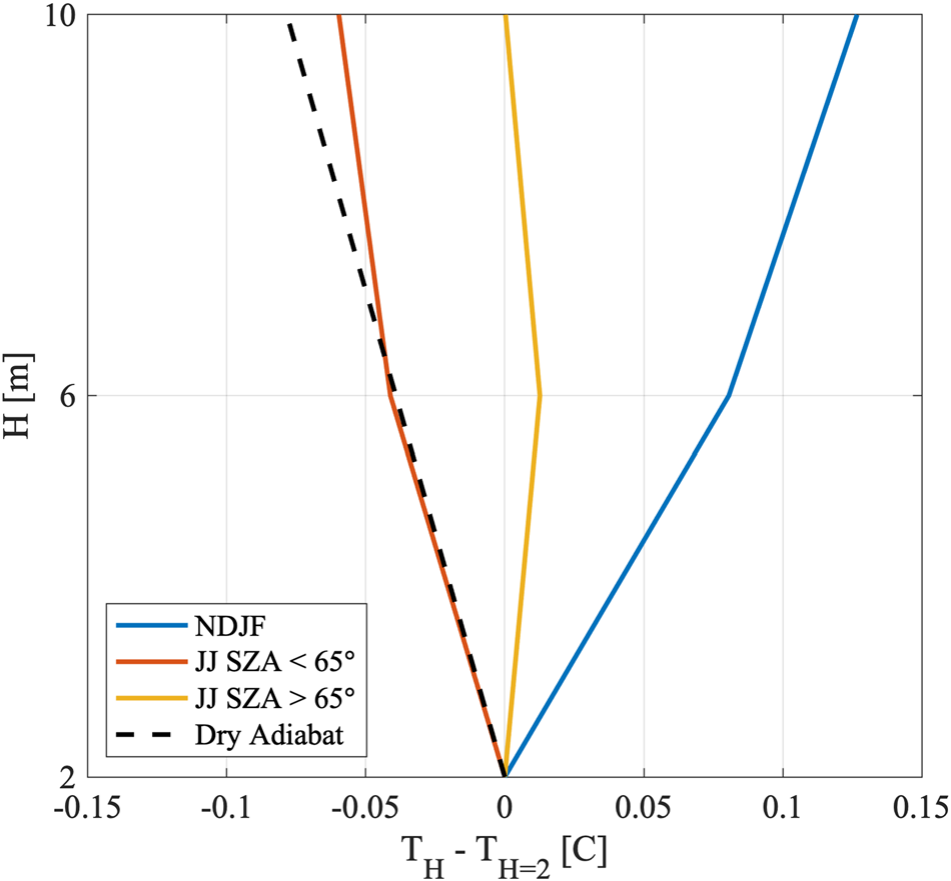
2-6-10 m temperature gradients relative to the temperature at 2 m height along the tower averaged for three different conditions: stable wintertime conditions (November-February) (blue); summertime (June-July) during the high solar zenith angle time of day (yellow); and low solar zenith angle time of the day (red). The dashed black line is the dry adiabat.

The uncertainty in the temperature measurement (Table 1) exceeds the typical lapse rate expected to be observed along the tower and mast. Thus, to facilitate the analysis of vertical gradients in temperature and humidity along the tower and mast, the relative calibrations between the sensors were adjusted using the 2 m instrument as a reference. Data were collected in the field while the tower was in the horizontal position (such that all sensors were ~1 m above the surface) over several days on three occasions distributed throughout the MOSAiC year: 19–25 October 2019; 24–28 June 2020; and 25–28 August 2020. On 4–9 December 2019, a spare HMT sensor and the WXT sensor from the fallen mast were both mounted near the 2 m sensor on the tower for additional intercomparisons. The collective data were used to determine offsets, which were < 0.1 C, except for the sensor used on the mast, which is less accurate and was adjusted by ~0.3 C. RH adjustments were < 1%. Thus, all adjustments were within the uncertainty of the measurement and serve to improve the relative accuracy between the sensors used on the tower without changing the absolute accuracy of any of the sensors. When it was determined to be necessary, to account for calibration drift and (seasonal) temperature dependency, the corrections were made variable as a linear function of time. The precise implementation of the corrections is as follows: (1) air temperature offsets were applied, (2) RH was recalculated using the adjusted temperature, (3) RH offsets were applied, and (4) adjusted RH and temperature were used to calculate other humidity variables (e.g., mixing ratio) supplied in the files. No adjustments were made to the ASFS meteorology. The offsets can be found in Table 4. Figure 16 shows examples of the gradients along the 10 m tower relative to the 2 m sensor after the adjustments were applied during strongly stable wintertime conditions (blue), weakly stable summertime conditions (yellow), and adiabatic summertime conditions (red).

**Table 4:** Offsets applied to tower and mast meteorology sensors.

Sensor S/N	Location	Data Period	Temp Offset (deg C)	RH Offset (%)
R218	Tower 2 m	10/19/19-9/20/20	dT = 0.00	dRH = −1.04
P197	Tower 6 m	12/4/19-9/20/20	dT = 7.5e-5 × YD - 0.13	dRH = +0.08
R217	Tower 6 m	10/19/19-12/9/19	dT = + 0.0972	dRH = −0.09
R428	Tower 10 m	10/19/19-9/20/20	dT = −0.000302 × YD + 0.17	dRH = + 0.07
WXT	Mast	10/19/19-5/2/20	dT = + 0.3380	dRH = −0.96

YD is the continuous year day where YD 1 is 1 January 2019. YD hence varies from 274 (1 October 2019) to 640 (1 October 2020. The application of the “dT” and “dRH” is such that the calculation/value was subtracted from the original measurement. These adjustments are applied in Level 2 and 3 but not Level 1.

## Data Availability

The code and associated libraries used to create Level 1, Level 2, and Level 3 processed files are based in Python with the following dependencies: Python > = 3.6; netCDF4 > = 1.3.0, NumPy > = 1.13.0, Scipy > = 1.1.0, Pandas > = 0.20, XArray > = 0.11; PVLib > = 0.8.1. Files uploaded to ADC have the following versions: Level 1 are v1.5 (1/8/2020) and Levels 2 and 3 v4.1 (2/1/2023). Code is archived on GitHub, https://github.com/MOSAiC-flux/data-processing.
